# Environmental Footprint of Wastewater Treatment: A Step Forward in the Use of Toxicological Tools

**DOI:** 10.3390/ijerph18136827

**Published:** 2021-06-25

**Authors:** Giorgio Bertanza, Jennifer Boniotti, Elisabetta Ceretti, Donatella Feretti, Giovanna Mazzoleni, Michele Menghini, Roberta Pedrazzani, Nathalie Steimberg, Chiara Urani, Gaia Claudia Viviana Viola, Ilaria Zerbini, Emanuele Ziliani

**Affiliations:** 1 DICATAM—Department of Civil, Environmental, Architectural Engineering and Mathematics, University of Brescia, Via Branze 43, I-25123 Brescia, Italy; giorgio.bertanza@unibs.it (G.B.); m.menghini@studenti.unibs.it (M.M.); 2 MISTRAAL Interdepartmental University Research Center—MISTRAL—Integrated Study Models for the Protection of Health and Prevention in Life and Work Environments, DSCS, Department of Clinical and Experimental Sciences, University of Brescia, Viale Europa 11, I-25123 Brescia, Italy; donatella.feretti@unibs.it (D.F.); giovanna.mazzoleni@unibs.it (G.M.); nathalie.steimberg@unibs.it (N.S.); chiara.urani@unimib.it (C.U.); 3 DSCS—Department of Clinical and Experimental Sciences, University of Brescia, Viale Europa 11, I-25123 Brescia, Italy; jenniferboniotti@yahoo.it; 4 DSMC—Department of Medical and Surgical Specialities, Radiological Sciences and Public Health, University of Brescia, Viale Europa 11, I-25123 Brescia, Italy; elisabetta.ceretti1@unibs.it (E.C.); gaia.viola@unibs.it (G.C.V.V.); ilaria.zerbini@unibs.it (I.Z.); 5 DIMI—Department of Mechanical and Industrial Engineering, University of Brescia, Via Branze 38, I-25123 Brescia, Italy; 6 DISAT—Department of Earth and Environmental Sciences, University of Milan—Bicocca, Piazza della Scienza 1, I-20126 Milano, Italy; 7 DICAr—Department of Civil Engineering and Architecture, University of Pavia, Via Ferrata 1, I-27100 Pavia, Italy; e.ziliani001@studenti.unibs.it

**Keywords:** activated sludge, baseline toxicity, carcinogenicity, endocrine disruption, genetic toxicity, mutagenicity

## Abstract

The assessment of the actual impact of discharged wastewater on the whole ecosystem and, in turn, on human health requires the execution of bioassays. In effect, based on the chemical characterization alone, the synergistic/antagonistic effect of mixtures of pollutants is hardly estimable. The aim of this work was to evaluate the applicability of a battery of bioassays and to suggest a smart procedure for results representation. Two real wastewater treatment plants were submitted to analytical campaigns. Several baseline toxicity assays were conducted, together with tests for the determination of endocrine activity, genetic toxicity and carcinogenicity of wastewater. A “traffic light” model was adopted for an easy-to-understand visualization of the results. Although the legal prescriptions of chemical parameters are fully complied with, bioassays show that a certain biological activity still residues in the treated effluents. Moreover, influent and effluent responses are not always appreciably different. Some tests employing human cells were revealed to be only partially adequate for environmental applications. An interesting and helpful development of the present approach would consist in the estimation of biological equivalents of toxicity, as shown for the estrogenic compound 17-β-estradiol.

## 1. Introduction

Water policies have been changing progressively during the recent decades, keeping up with the awareness of the need to save and preserve this resource.

The protection of water resources (in terms of volume and quality) goes far beyond mere compliance with the existing legal limits and guidelines: a radical change of perspective has been increasingly needed, leading to water resources being considered as part of complex ecosystems, where abiotic factors and biotic components coexist. Consequently, the entire supply chain, from the catchment of water for human consumption to its discharge into the environment, is experiencing progressive improvements, with the final aim to decrease the anthropogenic impact on waterbodies.

From this point of view, a careful and deep evaluation of potential effects of the Waste Water Treatment Plant (WWTP) effluents discharged into surface water, on both ecosystem and human health is of primary importance. WWTPs, in effect, may represent hotspots for those trace pollutants collected by sewers: the upgrading and development of efficient treatment technologies and the implementation of adequate process schemes represent the first response to this issue. The choice of the optimal solutions, however, must be based on the knowledge of the real impact of a plant [1,2,3].

It is widely recognized that a wastewater characterization focused only on chemical analyses provides a partial representation of the effects of a WWTP effluent. Several studies, indeed, have shown that only an integrated monitoring based both on chemical analyses and biological assays can yield to a more realistic evaluation of sewage degradation and detoxification performance of a WWTP [4,5,6,7]. In particular, testing an effluent as a whole, via the exposure to particular organisms allows overcoming the limitations caused by the very well-known phenomena of “something-from-nothing” and “a-lot-from-a-little”. These events are due to the possible compresence of analytes with different modes of toxic actions, also at concentrations lower than those causing quantifiable effects. Beside these conventional tests commonly prescribed by the international and national laws, deeper insights include the investigation of initiating key events, consisting in the very first interactions of chemical stressors with specific targets. By this way, major health-relevant toxicity pathways (e.g., endocrine disruption, tumor promotion) can be evidenced, even in the awareness that early repairing mechanisms might take place.

The aforementioned statements, which are still valid for mixtures, are even more important in case of wastewater treatment processes, where physical, chemical and biological transformations and decompositions lead to the production of new substances.

The results of an integrated monitoring, based on chemical analyses and toxicological tests, could be successfully integrated in the environmental footprint evaluation based on a Life Cycle Assessment (LCA) approach, which represents a useful tool for evaluating and benchmarking the actual impact of products and organizations [8,9].

Notwithstanding the general scientific consensus about the invaluable role of bioassays in environmental toxicity assessment, and the host of projects/studies/working groups (e.g., Tox21 [10]; EUTox-Risk [11]; the OECD platform [12]; SeqAPASS [13]), there is still an open debate about the choice of the most suitable tests, to be performed each time, also because of the lack of experience regarding the applications on real WWTPs.

The main aim of this work is to contribute to cover the gap of knowledge caused by the missed application of (existing or to be adjusted) bioassays, which are shown to be a powerful tool for understanding the real interactions of matrices discharged into the environment. Even more importantly, an attempt for overcoming ambiguity and misinterpretation of the results is presented. Common toxicological tools, such as tests performed on crustaceans, algae and luminescent bacteria, have been designed for evaluating effluent quality for legal purposes. The assessment of the real impact of wastewater discharge, however, should include a wide range of different biological target [14]. Therefore, a clear, broad and univocal synthetic way to represent ecotoxicological results should be developed. In this work, an integrated monitoring approach, using complementary chemical analyses and biological assays, was applied on two different WWTPs (one of these tested under two different operative conditions). Experimental results were processed and a “traffic light” easy-to-read representation was proposed.

## 2. Methods

### 2.1. The Studied WWTPs

The municipal WWTPs chosen as case studies are located in the North of Italy. WWTP A (design size 370,000 p.e.) treats domestic wastewater with a remarkable contribution of industrial discharge from the agro-food sector. WWTP B (design size 60,000 p.e.) treats important amounts of winery effluents, which, during the grape harvest time (September and October), increase the pollutant influent loads, respect to the “routine” period. Both plants adopt the conventional activated sludge process. A detailed description is reported in the following paragraphs (see also additional information in Table S1 of Supplementary Material).

#### 2.1.1. WWTP A

WWTP A consists of: pre-treatments (coarse screening, fine screening, grit and oil removal), primary settling (10,400 m^3^), pre-denitrification (7140 m^3^), oxidation/nitrification (16,660 m^3^), secondary settling (26,140 m^3^). Excess sludge undergoes dynamic thickening, anaerobic digestion and mechanical dewatering. The typical influent flowrate is equal to 74,900 m^3^/d (median value of the years 2013–2017; standard deviation is 8800 m^3^/d). Unlike the flowrate, the wastewater composition varies during winter and summer periods, due to the contribution of the seasonal industrial activities (agro-food manufacturing sector). Typical concentrations of the main pollutants are the following, in the summer and winter periods, respectively. Standard deviations are reported between brackets. COD: 300 (42) and 540 (85) mg/L; BOD_5_: 170 (30) and 300 (63) mg/L; total nitrogen: 45 (8) and 70 (12) mg/L; total phosphorus: 5.0 (0.8) and 7.2 (1.4) mg/L. Effluent concentrations are rather stable all along the year as show by the following data. COD: 22.7 (8.0) mg/L; BOD_5_: 5.8 (2.5) mg/L; total suspended solids: 8.5 (5.8) mg/L; total nitrogen: 14.5 (5.6) mg/L; total phosphorus: 1.5 (0.9) mg/L. The sludge retention time in the aerated reactor is equal to 8 ± 2 d; wastewater temperature ranges between 15 and 26 °C (industrial discharges prevent the temperature to drop below 15 °C in wintertime).

#### 2.1.2. WWTP B

The WWTP B treatment train includes the following units: equalization (1300 m^3^), preliminary treatments (coarse screening, fine screening, grit and oil removal), pre-denitrification (2160 m^3^), oxidation-nitrification (7120 m^3^), final sedimentation (4564 m^3^), disk filtration, UV disinfection. The sludge treatment line consists of dynamic thickening and mechanical dewatering. The main influent characteristics in the routine and grape harvest periods, respectively, are reported in the following (standard deviations between brackets). Flowrate: 20,300 (2500) and 26,170 (3100) m^3^/d; COD: 290 (32) and 430 (75) mg/L; BOD_5_: 130 (18) and 220 (40) mg/L; total nitrogen: 17 (2.0) and 18 (1.9) mg/L; total phosphorus: 2.5 (0.3) and 3.3 (0.4) mg/L. As for the effluent, the typical characteristics of the two periods are the following. COD: 17.3 (5.2) and 19.9 (6.3) mg/L; BOD_5_: 6.9 (3.2) and 8.0 (4.1) mg/L; total suspended solids: 6.9 (5.3) and 7.3 (6.0) mg/L; total nitrogen: 5.8 (3.0) and 5.0 (3.2) mg/L; total phosphorus: 0.5 (0.3) and 0.4 (0.3) mg/L. The sludge retention time is kept between 15 and 25 days in aerated reactors. Wastewater temperature ranges between 10 and 25 °C.

### 2.2. Sampling Procedure

Three monitoring campaigns were conducted, by sampling influent and effluent wastewater (Figure S1): a single campaign in case of plant A and a double survey in case of Plant B, in order to include both grape harvest time and routine operation. Sampling represents a crucial step in the characterization of different streams, hence in the evaluation of plant performance. Several authors have underlined the huge variability of trace pollutants content throughout a day or a week: influent and effluent single grab samples are not representative at all of the actual trends (see, inter alia, [15,16,17,18]). For these reasons, the duration of every monitoring campaign was set at two weeks, much longer than the hydraulic retention time (HRT). Twenty-four hour flow-proportional composite samples were collected daily, at each sampling point, in refrigerated auto-samplers, equipped with Teflon pipes and dark glass containers. In order to obtain one sample, which was representative of the whole monitoring period, to be submitted to bioassays, daily samples were finally mixed together. Based on previous research experiences of the authors (see for instance [4,5,19]), a cumulative volume of at least 36 L/sample was collected, for performing both chemical analyses and biological tests. Some analyses required sample pre-treatment immediately after collection, as described in detail in Chapter 14 of Ecotoxicological QSARs book [20].

### 2.3. Selection of the Bioassays

The tests were chosen based on their standardization, high reproducibility, automated protocol, sensitivity, adequacy, statistical robustness, biological representativeness, possibility of extrapolating the in vitro results to potential in vivo hazards, possibility of cross-species extrapolation. Then, acute and chronic toxicity were taken into account, by selecting endpoints, linkable with short and long time effects (up to transgenerational events). Moreover, different levels of biological organization were targeted (organisms, tissues, cells) as well as different biological complexity (prokaryotes, eukaryotes; animals, plants; unicellular, multicellular). Finally, the bioassays can detect both baseline toxicity and particular modes of action, adopted in order to correlate key events with biological answers. The modes of action (MOA) explored in this toxicological study, as well as the specific tests and the measured phenomena are the following:

- baseline toxicity: green alga growth inhibition [21]; marine bacteria bioluminescence inhibition [22]; freshwater cladoceran mobility inhibition [23]; plant roots growth inhibition [24]; neutral red dye uptake by viable cells [25]
- endocrine disruption: luciferase activity quantification in human breast cancer cell line [19]
- genetic toxicity: point reverse mutations in bacteria [26,27]; chromosomal mutation in plant roots cells [28,29]; Single Cell Gel Electrophoresis on human leukocytes [26,30,31]
- carcinogenicity: number of malignant foci or transformed cells [32,33,34]; gap junction-mediated intercellular communication [35]

The experimental methodologies are described at length elsewhere [20].

### 2.4. Chemical Analyses

Chemical analyses were addressed to the determination of conventional parameters (for assessing the general plant performance) and the quantification of inorganic and organic pollutants, to better characterize the quality of the streams. Selected conventional parameters are: total suspended solids, biochemical oxygen demand (BOD), chemical oxygen demand (COD), total nitrogen, total phosphorus. These pollutants were analyzed daily. Boron, vanadium, chromium, manganese, iron, nickel, copper, arsenic, selenium, cadmium, antimony, aluminum, mercury, lead were analyzed weekly. Herbicides, insecticides and degradation by-products, perfluorinated alkyl substances, polynuclear aromatic hydrocarbons were also determined once a week. Details of analytical methods are reported in Supplementary Material (Table S2).

### 2.5. Data Processing

The integrated and overall examination of the results requires data harmonization, in order to have a comprehensive view of possible toxic effects exhibited by the tested samples. Thus, a “traffic light” visualization was proposed. A color (green, yellow or red) was attributed as a function of biological response intensity: the criteria underpinning the threshold settings are widely explained in Supplementary Material. Table 1 reports a brief description of the meaning of the chromatic code for each test, as well as the type of sample to be considered (e.g., raw undiluted or an extract).

An important information to take into account for the correct interpretation of results is the correlation between the biological response and the degree of dilution (for those test conducted on raw samples) or concentration (when extracts are used) of the samples. In case of dilution of the raw wastewater the calculation is quite simple. On the contrary, when sample pre-treatment is required, both dilution and enrichment processes (the latter consisting in a solid phase extraction—SPE) must be taken into account.

In this case, the range of sample concentrations tested in the different bioassays was expressed in unit of Relative Enrichment Factor (*REF*) [36,37], which is the combination of the enrichment factor of the SPE process (*EF_SPE_*) and the actual sample dilution in the bioassay (dilution factor: *DF_bioassay_*) (Equation (1)).

(1) $$REF=EF_{SPE}\cdot DF_{bioassay}$$

The *EF_SPE_* was calculated using Equation (2) as the ratio between the volume of processed water (*V_water_*) to the volume of resulting extract in solvent (*V_extract_*).

(2) $$EF_{SPE}=\frac{V_{water}}{V_{extract}}$$

The dilution factor of each tested dose was calculated using Equation (3).

(3) $$DF_{bioassay}=\frac{V_{extract\;added\;to\;bioassay}}{V_{bioassay}}$$

Thereby, a value of *REF* equal to 1 (REF 1 sample) means that organic analytes concentration in the bioassay is equivalent to that of the unprocessed water (devoid of metals, inorganic anions and a fraction of colloidal organic, after solid phase extraction). On the contrary, a value higher, or lower, than 1 expresses, respectively, a sample concentration or dilution.

The values of *EF_SPE_* and the final volumes considered for each bioassay are reported in Table 2.

## 3. Results and Discussion

### 3.1. WWTP A

#### 3.1.1. Chemical Analyses

The main influent and effluent characteristics are summarized in Table 3. WWTP A achieves high organic removal efficiencies (COD removal greater than 95%; BOD removal greater than 98%) as well as a remarkable removal of nitrogen (74%) and phosphorus (73%). The separation of suspended solids in the final sedimentation tanks is also very effective (very low effluent concentration).

Table 4 shows that measured metals and semimetals effluent concentrations are well below the respective discharge standards (Legislative Degree 152/2006 [38]) as well as the EC_50_ values reported in literature for *D. magna*. Only for copper and mercury the measured concentrations are relatively close to the respective EC_50_.

During the monitoring campaign, all the measured polynuclear aromatic hydrocarbons, chlorinated insecticides and herbicides were below their detection limits, which are reported in Table S3 of Supplementary Material. Conversely, perfluorinated alkyl substances were detected at the concentrations reported in Table 5. As it can be seen, only pefluorohexanoic acid and perfluoroctanoic acid were detected above the respective limit of quantification (LOQ): therefore, the sums of congeners and isomers reported in Table 5 are given only by the concentrations of these two molecules. Interestingly, they are widely below the environmental quality standards (EQS) proposed by the Water Framework Directive 2000/60 [42]: 1 µg/L for PFHxA and 0.1 µg/L for PFOA.

#### 3.1.2. Baseline Toxicity

Baseline toxicity exerted on daphnids, algae and bacteria was not decreased after the treatment: detailed results of these bioassays are reported in the Supplementary Material (Table S4), while their translation into the proposed chromatic code is explained in Section 3.1.6, where this phenomenon appears evident.

The *Allium cepa* test showed toxicity in influent undiluted sample: the roots elongation was negatively influenced (red light, as described in Section 3.1.6) and the 1:2 dilution corresponded to the EC_50_ (see Table S5). The effluent undiluted sample did not inhibit the lengthening of the roots (see Figure S2) and no signs of toxicity were observed (green light). Therefore, the subsequent genotoxicity tests on effluent (vide infra) could be performed using the undiluted and diluted samples (1:2, 1:10, 1:100), while the influent was assayed using only diluted samples (1:2, 1:10, 1:100) due to the presence of toxicity.

Cell toxicity was assessed on hepatic cells because of their sensitivity to xenobiotics. Neutral Red assay was chosen because of its sensitivity for detecting cell homeostasis. The MTT assay [20] was excluded because almost unsensitive (data not shown) in tested experimental conditions.

**Figure 1:**
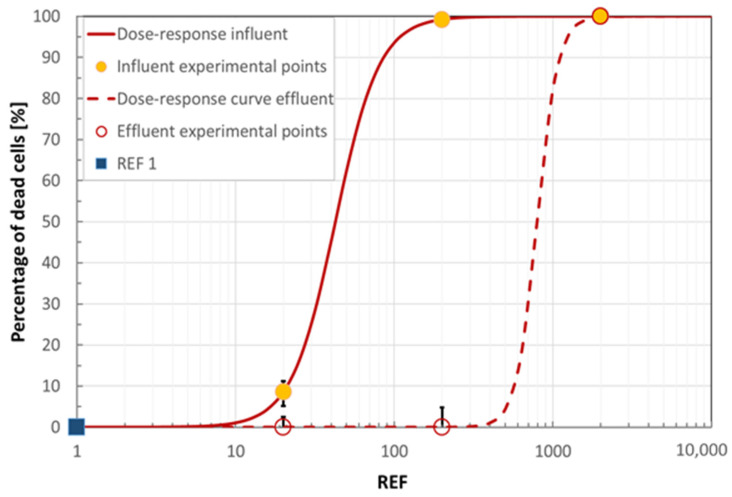
Cell viability in IAR203 hepatic cells exposed to different concentrations of influent and effluent wastewater extracts. Values are expressed as % versus the untreated (negative) control. Cell viability was assessed with Neutral Red Assay in triplicate: error bars show maximum and minimum values.

As shown in Figure 1, a typical dose-response curve (obtained with normalized value: see Supplementary Material) was defined by testing extracts at different REFs, the lowest doses (REF 20) resulting negligibly or not cytotoxic at all for the influent and effluent samples, respectively. IC_50_ (concentration which gives 50% inhibition of cell growth) for influent is twenty times higher than for the effluent, demonstrating the positive effect of the plant treatments on cell viability and thus the noteworthy reduction of toxic potential of this wastewater. The REF 1 effect was extrapolated by dose-response curves: no toxicity was estimated (green color, as detailed in Section 3.1.6).

#### 3.1.3. Estrogenic Activity

##### ERE-tk_Luc_MCF-7 Test

**Figure 2:**
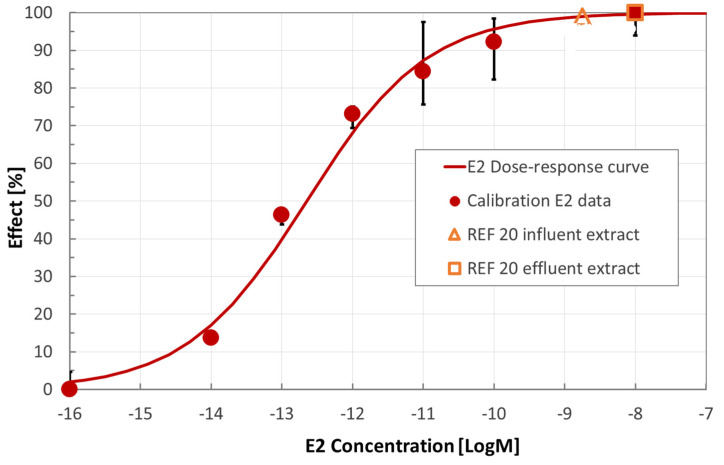
Estrogenic activity was assessed on MCF-7 mammary cells exposed to different concentrations of E2 and compared with REF 20 samples of influent and effluent wastewater. The assay was conducted in triplicate: error bars show maximum and minimum values.

Estrogenicity of wastewater was assessed on ERE-tkLuc mammary cells sensitive to estrogens. A non-cytotoxic dose of diluted wastewaters (REF 20) was used. Using the standard curve elaborated with 17β-estradiol (E2) (see Table S6), the endocrine disrupting activity of influent and effluent wastewaters was determined. As shown in Figure 2 (curve dose-response obtained with normalized values as explained in Supplementary Material), the estrogenicity of the REF 20 wastewater extract was not reduced by the polishing treatment. The mixture of estrogen-like compounds present in the REF 20 extract have an overall activity equivalent to the one exerted by E2 at the highest tested concentrations. The endocrine disruption effect of undiluted effluent discharge (REF 1), extrapolated by the effect of the REF 20 extract, was expressed as E2 equivalent concentration. It yielded an E2 biological equivalent concentration equal to 136 ng/L, which, according to [37], can be compared to the trigger value (0.2 ng/L). This comparison would indicate a rather bad quality of the effluent wastewater in terms of estrogenicity. Nevertheless, it is worth to be noted that the trigger value was derived from the Australian Guidelines for Water Recycling Augmentation of Drinking Water Supplies (AGWR). Indeed, the present study refers to the discharge in a surface waterbody, namely a river. Therefore, a direct comparison would not be appropriate. Similarly, just as a reference, the threshold for drinking water reported in the WHO Guidelines [43] is equal to 1 ng/L, again much lower than the equivalent E2 concentration of the WWTP effluent.

#### 3.1.4. Genetic Toxicity

##### Ames Test

The results of Ames test, expressed as mutagenicity ratio (MR), are presented in Table 6. According to the rules in Table 1, no samples showed mutagenic activity on *Salmonella,* in both strains, with and without exogenous metabolic activation (green light for all samples, in Table 7). In effect, MR values close to 1 indicate no differences respect to the negative control. The more the values approach 0, the more they indicate toxicity: both samples showed high toxicity, indeed, in particular the influent. The high toxicity could have masked possible mutagenic effects preventing bacteria growth.

##### Comet Test

**Figure 3:**
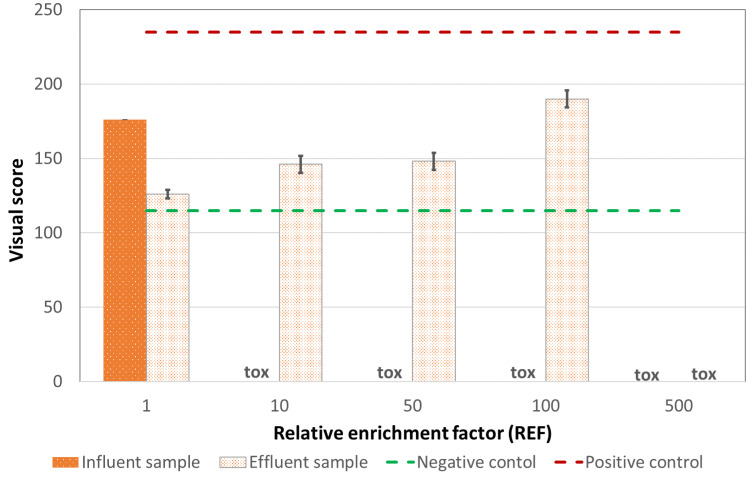
Visual classification of DNA damage, expressed as “visual score”, at increasing concentration. The error bars show the standard deviation.

The results of comet assay are reported in Figure 3 and Table S7 (Supplementary Material). Both samples caused a significant increase of DNA strand breakage in human leukocytes. Influent wastewater showed genotoxic activity, both versus the negative control and the effluent, even though the test was largely affected by toxicity, starting from very low doses (red light was then attributed). A significantly increased genotoxicity, compared to controls, was found in wastewater effluent, where a dose-response curve is evident (red light was attributed also to the effluent). Again, these samples exhibited toxicity, though only at the highest dose. Toxic effects in the comet assay were mainly represented by the preponderant presence of the so-called ‘hedgehogs’, corresponding to nucleoids with small or non-existent heads and large, diffuse tails that are assumed to represent apoptotic/necrotic cells.

##### Allium Cepa Genotoxicity Test

As shown in supplementary material (Tables S8 and S9), no genotoxicity was observed in *Allium cepa* roots at all tested dilutions (green light for all samples in Table 7). Again, toxic effects have been observed in the influent, at 1:2 dilution only.

#### 3.1.5. Carcinogenicity

##### Tumor Promotion

**Figure 4:**
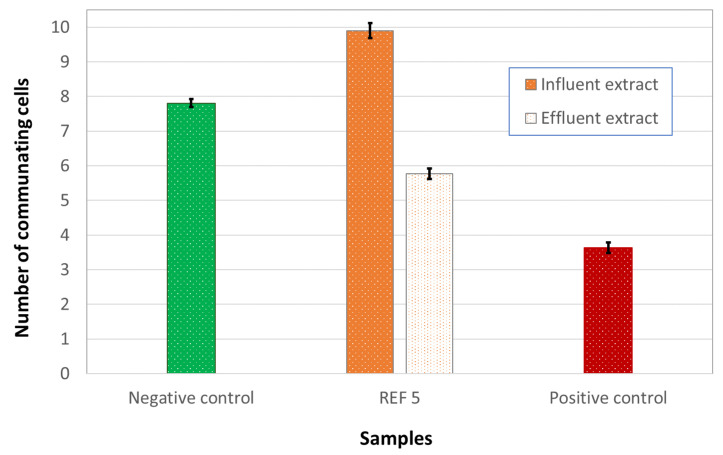
Gap junction Intercellular communication was assessed in IAR203 hepatic cells exposed to influent and effluent wastewaters. The error bars show the standard error of mean.

The tumor potential of wastewater was assessed on IAR203 hepatic cells, that present a high communicating capacity. A non-cytotoxic dose of extracted wastewaters (REF 5) was used. Beside the negative control, cells were treated with TPA (positive control), a well-known inhibitor of communication mediated by gap junction (GJ) and a reference tumor promoter. As shown in Figure 4 whereas the influent did not inhibit intercellular communication, the effluent acquired an evident inhibiting effect on gap junctions, though lower than the positive control.

#### 3.1.6. Traffic-Light Coding of Toxicological Data

Table 7 displays the chromatic attributions given to toxicological results, according to the criteria explained in section “2.5 Data processing” and Table 1.

First of all, the importance of using different tests, within the same mode of toxic action, has to be underlined. In effect, responses to different organisms should be compared together, to obtain reliable results. As for baseline toxicity, for example, two tests (namely *A. fischeri* and *R. subcapitata*) show an apparent worsening of the effluent, with respect to the untreated wastewater. Indeed, the quantified bacteria luminescence inhibition passed from 48% (influent) to 56% (effluent), which is a rather small or even not significant difference, considering the uncertainty of the bioassay. Likewise, and even less significantly, the algal growth inhibition increased from zero to only 11% (10% being the minimum threshold causing the yellow color attribution). On the contrary, the *A. cepa* assay shows an appreciable quality improvement, corresponding to a two-classes jump (from red to green) after biological treatment. It is interesting to compare these results with legal limits established for defining an effluent as acceptable. In particular, the Italian regulation states that less than 50% immobilization/inhibition is to be caused (tests on crustaceans, luminescent bacteria and green algae). Under this perspective, only the bacteria reveal particularly sensitive, after exposure to both influent and effluent, whose effects remain almost unchanged after the treatment. On the contrary, crustaceans and green algae are not significantly affected, thus describing good quality samples.

The same level of estrogenicity was observed for the influent and effluent extracted samples red color was attributed in both cases. Nevertheless, it has to be underlined that samples were highly concentrated (REF 20). Indeed, the estimated equivalent E2 concentration of the REF 1 sample resulted much higher than the reference values for groundwater recharge and drinking water. And this is very interesting considering that measured organic pollutants concentrations were extremely low.

Regarding genetic toxicity, all performed tests show no difference between the influent and effluent samples. In particular, green color has been always attributed, apart from the case of comet assay. Interestingly, the damage occurred to DNA of human leukocytes might be reassessed based on the inherent role played by this assay for the environmental impact assessment. Indeed, the comet test does not allow detection of the DNA fragments, which originate from apoptosis and necrosis: therefore, cytotoxicity may possibly lead to false positive and negative results (the cytotoxic effect at the highest doses, both for influent and effluent appears clearly when the chromatic code is used for describing the biological behaviors: see Table 7 and Section 3.2.6). Further investigations should include biological systems which are more adequate for ecological monitoring, such as freshwater mollusks (as *Perna viridis* and *Corbicula fluminea*). Several species, in addition, could be employed in a passive monitoring, placed in situ, for instance downstream the effluent discharge point. The exploitation of Comet test as ecotoxicological tool must overshoot the interpretative criteria of the biomedical research and human toxicology; proper models, chosen based on ecological roles and life cycle (stages), would provide more accurate information on freshwater ecosystem impacts.

Finally, gap junctions appear to be slightly inhibited only in the effluent samples. As discussed above for *A. fischeri* and *R. subcapitata,* the yellow color was attributed being the result on the borderline between green and yellow class. Consequently, the difference between the influent and the effluent has to be considered as not relevant.

In short, notwithstanding the legal requirements compliance of the effluent for the chemical parameters, a certain level of residual toxicity is detected in prokaryotes and human cells (modes of action: endocrine disruption and genetic toxicity). Surprisingly, influent and effluent samples did not show appreciably different behaviors.

### 3.2. WWTP B

#### 3.2.1. Chemical Analyses

The main influent and effluent characteristics are summarized in Table 8. In both monitoring periods the WWTP B achieves high organic and nutrient removal efficiencies (almost 94% for COD, between 94 and 97% for BOD, between 66 and 80% for nitrogen, 93% for phosphorus) and shows a very good performance of final sedimentation (see the total suspended solid concentration in the effluent). Additionally, in the routine period, the influent surfactant concentration was 11 mg/L, more than one order of magnitude higher with respect to the grape harvest time (0.4 mg/L).

As for WWTP A, the metals and semimetals effluent concentrations are widely below the respective discharge standards (Legislative Degree 152/2006), as well as the EC_50_ towards *D. magna* (see Table 9). As in the previous case, only copper and mercury concentrations approach the respective EC_50_ values for *D. magna*.

Table 10 reports the organic pollutants, which resulted above their detection limits, namely perfluorinated alkyl substances and two herbicides. The complete list of the quantified analytes, with the respective LOQs, is reported in Supplementary Material (Table S10). The environmental quality standards proposed by the Water Framework Directive 2000/60 [42] for terbutryn (0.065 μg/L), PFHxA (1 µg/L), and PFOA (0.1 µg/L) are complied with. Additionally, metolachlor is lower than 0.1 μg/L, which is the yearly average threshold for any single unspecified pesticide defined by the Italian legislative decree 172/2015 (implementation of the European Directive 2013/39/EU).

#### 3.2.2. Baseline Toxicity

All the conventional baseline toxicity tests prescribed by the law demonstrate the compliance of the effluent in both campaigns (detailed data in Table S11 of Supplementary Material). Moreover, the treatment generally improved the quality of wastewater, as it appears from the summarization of each result, according with the chromatic code (Section 3.2.6), as shown by most of the performed tests. Actually, *R. subcapitata* test shows a different result for the first campaign samples; notably, this is the only pejorative result out of six, thus not modifying the overall judgement. It can be observed that the influent wastewater toxicity measured during the second campaign is higher. One reason could be the relevant concentration of surfactants, whose toxic action is known [44].

Influent and effluent tested on *Allium cepa* exhibit no toxicity in both samples of the first campaign (green light). Conversely, during the spring campaign, the influent sample showed a slight toxicity (yellow), which disappeared after biological treatment (green) (see Table S12 and Figure S3). The subsequent genotoxicity tests were then carried out on the undiluted and diluted samples (1:2, 1:10, 1:100).

**Figure 5:**
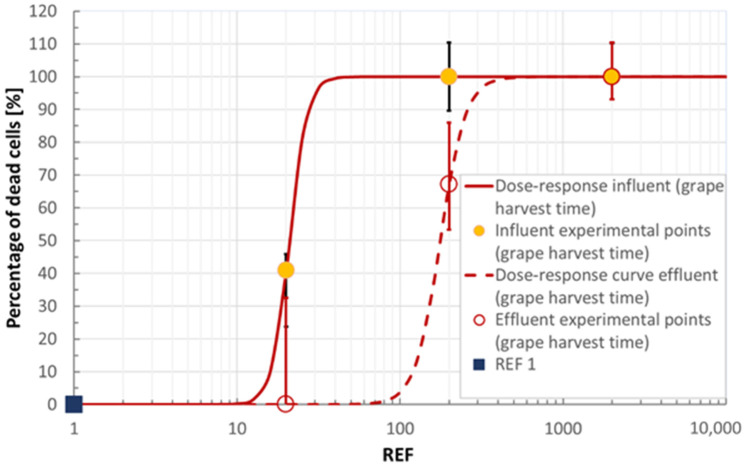
Cell viability in IAR203 hepatic cells exposed to different concentrations of influent and effluent wastewaters (first campaign). Values are expressed as % versus the untreated control. Cell viability was assessed with Neutral Red Assay in triplicate: error bars show maximum and minimum values.

**Figure 6:**
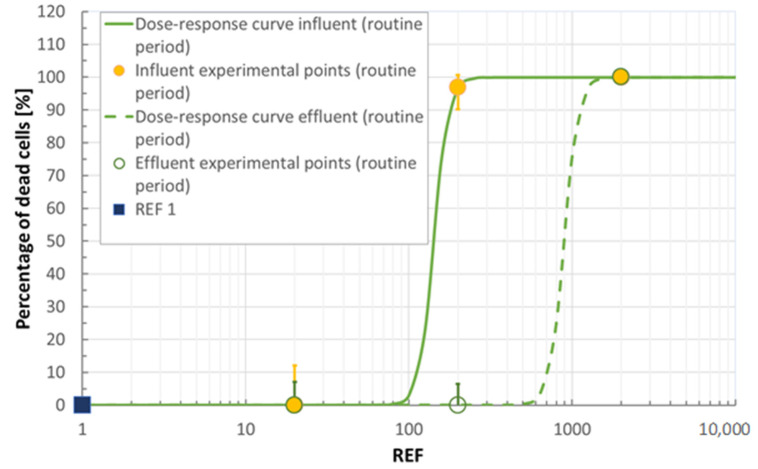
Cell viability in IAR203 hepatic cells exposed to different concentrations of influent and effluent wastewaters (second campaign). Values are expressed as % versus the untreated control. Cell viability was assessed with Neutral Red Assay in triplicate: error bars show maximum and minimum values.

Cytotoxicity (MTT test carried out on IAR203 hepatocytes with REF 1 sample) reveals absent in both periods, thus the green color was attributed. Nevertheless, as shown in Figure 5 and Figure 6 (where the experimental values are normalized as explained in Supplementary Material), the IC_50_ for the influent is about ten times higher than for the effluent, demonstrating the positive effect of the polishing treatment. Indeed, the IC_50_ is achieved only at very high concentration factors, while the chromatic evaluation refers to the REF 1 condition.

#### 3.2.3. Estrogenic Activity

##### ERE-tk_Luc_MCF-7 Test

**Figure 7:**
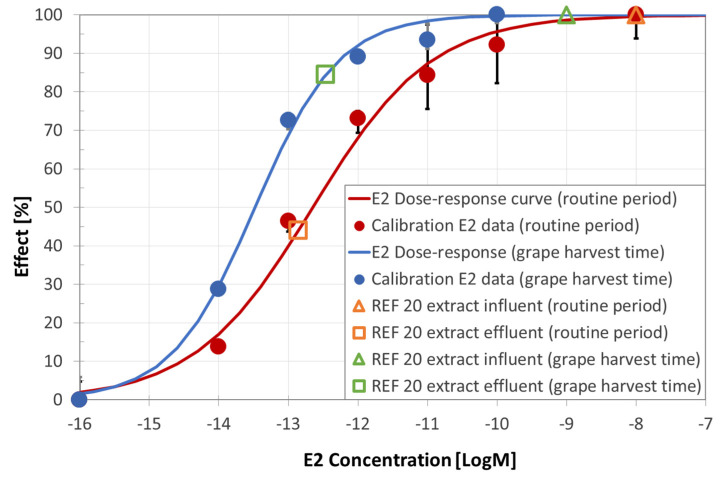
Estrogenic activity was assessed on MCF-7 mammary cells exposed to different concentrations of E2 and compared with REF 20 samples of influent and effluent wastewater of each campaign. The assay was conducted in triplicate: error bars show maximum and minimum values.

As mentioned above, a non-cytotoxic dose of extracted wastewater (REF 20) was used for assessing the estrogenicity. Using the standard curve (normalized values as explained in Supplementary Material) elaborated with E2 (see Table S13), the endocrine activity of samples was determined. As shown in Figure 7, plant B, unlike plant A, reduced the estrogenicity, at a greater extent during the routine period, thus leading to a chromatic change (yellow vs. red: see Section 3.2.6). The positive effect of treatments is also demonstrated by calculating the REF 1 E2 equivalent concentration (0.0047 ngE2/L and 0.0019 ngE2/L, for the grape harvest and routine period, respectively). These equivalent concentrations are much lower than the respective trigger value (0.20 ngE2/L) reported in Escher et al. [37].

#### 3.2.4. Genetic Toxicity

##### Ames Test

The results of Ames test, expressed as mutagenicity ratio, are presented in Table 11.

A slight mutagenic effect was displayed by the TA98 strain without the exogenous metabolic activation (S9), revealing the presence of directly active mutagens causing frameshift mutation, also indicating the detoxifying action of the S9 [45]. For this reason, the yellow color was attributed in Table 12.

*Salmonella typhimurium* strains treated with and without enzymatic activation have a similar behavior in terms of genetic damages. Anyway, influent taken during grape harvest time exhibits a slightly weaker toxicity with respect to other samples.

##### Comet Test

**Figure 8:**
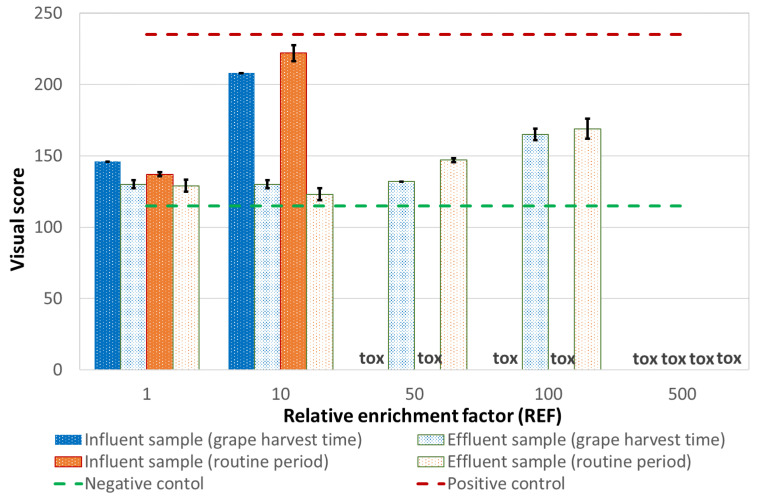
Visual classification of DNA damage, expressed as “visual score”, at increasing concentration. The error bars show the standard deviation.

All the samples collected at the WWTP B show damages to the DNA of exposed cells leading to red color attribution (see Table 12). Effluent exhibited baseline and genetic toxicity to a lesser extent respect to the influent, significantly genotoxic, therefore, causing a damage comparable to the positive control (see Table S14). The two campaigns yielded similar results. DNA damages were still higher with respect to the negative control after the biological treatment, as shown in Figure 8.

##### Allium Cepa Test

A slight increase in chromosomal aberrations was observed in undiluted wastewater before the biological treatment in autumn sample only (yellow light), using *Allium cepa* genotoxicity tests (4.4% vs. 2.5% negative control and 3.3% effluent). No other sample was able to induce DNA damage (green light), as either chromosomal aberrations or micronuclei (as reported in Supplementary Material, Tables S15 and S16).

#### 3.2.5. Carcinogenicity

##### In Vitro Cell Transformation Assay

As for the other tests, the in vitro transformation assay was performed after the preliminary cytotoxicity assessment, as detailed in [32].

**Figure 9:**
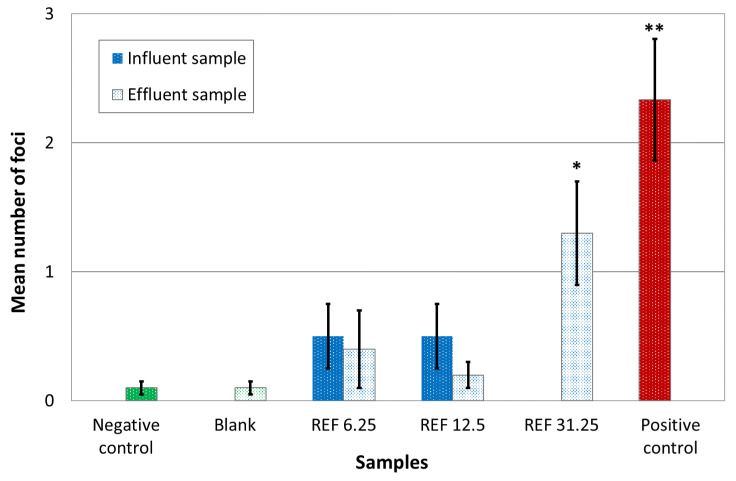
Mean number of transformed foci formed at the end of the cell transformation assay after treatment with non-cytotoxic dilutions of wastewaters, and with positive control (3-MCA). * *p* < 0.05, ** *p* < 0.01.

The mean number of transformed foci (Type II and III) generated after the exposure to the samples is shown in Figure 9, where a significant difference (*p* < 0.05) is clear in samples at REF 31.25. The significance threshold (*p* < 0.05) suggests a yellow level of attention (see Table 12). Whereas, as expected, the number of foci formed after exposure to the positive control (3-MCA) evidenced a statistically significant difference with *p* < 0.01.

##### Tumor Promotion

**Figure 10:**
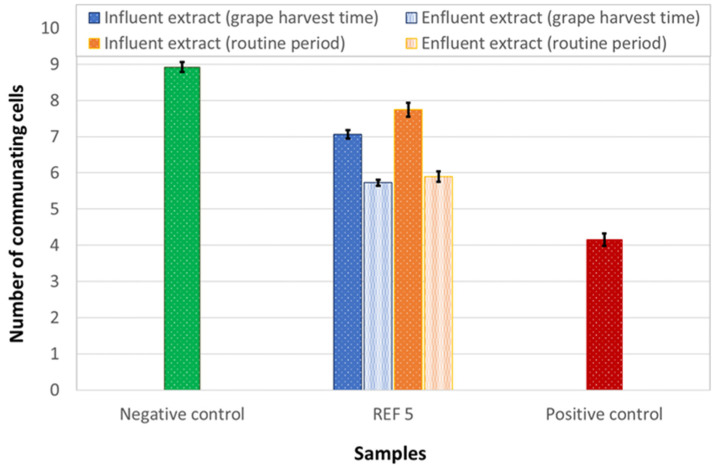
Gap junction Intercellular communication was assessed in IAR203 hepatic cells exposed to influent and effluent wastewaters. The error bars show the standard error of mean.

As shown in Figure 10, whereas the influent only slightly inhibits cell communication, the effluents present a more significative inhibiting effect of gap junctions, although not so marked as that induced by TPA (positive control).

#### 3.2.6. Traffic-Light Coding of Toxicological Data

The translation of all the results into a chromatic code is displayed in Table 12. No significant differences can be generally observed between the biological responses of the influent and effluent samples, given that a double quality class jump never occurs. Anyway, effects measured by several bioassays, such as *D. magna* test, *A. fischeri* test, *A. cepa* and MCF-7 cells estrogenicity decrease, at least for one campaign, after the wastewater treatment. On the contrary, other assays, such as *R. subcapitata* test, Ames test, CTA and tumor promotion test show, in some cases, a more toxic effect of the effluent sample. Interestingly, the influent wastewater of the routine period induces a greater baseline toxicity, partially ascribed to the high concentration of surfactants. It has to be underlined, however, that the results marked with red Color refer to experiments conducted on heavily concentrated samples. Indeed, as for the MCF-7 test, the estimated equivalent E2 concentration of the raw sample is very low. Moreover, regarding the Comet test, the statements reported above on the adequacy and suitability of this biological model (namely, the human leukocytes) is now under debate.

To sum up, the quality of the effluent can be considered as acceptable, in terms of legal prescriptions and based on the results of the majority of the proposed tests. As observed for WWTP A, only the response of human cells leads to apparent criticalities, possibly due to the partial adequacy of this biological model for environmental applications. A substantial improvement of effluent quality is obtained after the treatment.

## 4. Conclusions

The findings obtained in this study show the opportunity to perform a series of diverse assays, given that several specific endpoints can be targeted, and different biological models chosen, thus enabling to get a possible confirmation of the results. Furthermore, some tests, as in the case of estrogenicity assessment, describe the overall behavior of mixtures of substances, which are considered with increasing attention by the policy makers and included in a growing number of legislative documents.

The power of a multitiered approach suggested by several researchers has been confirmed, although further monitoring campaigns on different wastewater treatment plants are required, in order to better tune the sensitivity of the experiments and to select new ones (either replacing or adding those proposed here), with particular focus on the need to diminish the masking effect exerted by toxicity towards definite endpoints. Additionally, some tests conducted on human cells (namely, the detection of estrogenic response of MCF-7 and of DNA fragmentation in leukocytes) revealed highly sensitive.

Specifically, the suggested traffic light approach might help in getting a comprehensive overview. Interestingly, bioassays revealed that, in some cases, the effluent maintains a certain biological activity.

The authors believe that the conventional toxicity assays should be profitably integrated with additional and complementary ones, to properly characterize the possible effects of the discharged wastewater on the freshwater biota. The estimation of biological equivalents of toxicity, obtained by testing reference substances, could provide further quantitative information, as shown in the case of the estrogenic compound E2.

The outcomes of the present research are very promising, though not exhaustive, and they represent a solid starting point for further studies, which are already ongoing.

## Figures and Tables

**Table 1:** Criteria adopted for color attribution to experimental results (E2 = 17β-estradiol; type of sample: REF = relative enrichment factor, as defined in the main text; RU = raw undiluted).

Mode of Toxic Action	Bioassay	Green	Yellow	Red
**Baseline toxicity**	*R. subcapitata* test	Effect measured on RU sample lower than 10%	Effect measured on RU sample ranging between 10% and 50%	Effect measured on RU sample higher than 50%
	*Aliivibrio fischeri* test	Effect measured on RU sample lower than 10%	Effect measured on RU sample ranging between 10% and 50%	Effect measured on RU sample higher than 50%
	*D. magna* test	Effect measured on RU sample lower than 10%	Effect measured on RU sample ranging between 10% and 50%	Effect measured on RU sample higher than 50%
	*Allium cepa* toxicity test	Effect measured (root length) on RU sample <40% respect to the negative control	Effect measured (root length) on RU sample ranging between 40% and 60% of the negative control	Effect measured (length reduction) on RU sample >60% respect to the negative control
	Neutral Red uptake assay	Effect exhibited on the REF 1 sample lower than the effect corresponding to IC_20_	Effect exhibited on the REF 1 sample ranging between the effects corresponding to IC_20_ and IC_50_	Effect exhibited by the REF 1 sample higher than the effect corresponding to IC_50_
**Endocrine disruption**	ERE-tk_Luc_MCF-7	Measured effect lower than the effect corresponding to the E2 EC_20_	Measured effect ranging between the effects corresponding to the E2 EC_20_ and EC_50_	Measured effect higher than the effect corresponding to the E2 EC_50_
**Genetic toxicity**	Ames test	The mutagenicity ratio is <2	The mutagenicity ratio is in between 2 and 2.5	The mutagenicity ratio is >2.5
	*Allium cepa* genotoxicity tests (CA, MN)	No statistically significant differences between the samples and the negative control (*p* > 0.05)	Statistically significant differences between the samples and the negative control (*p* < 0.05)	Statistically significant differences between the samples and the negative control (*p* < 0.05, *p* < 0.01, *p* < 0.001) and effects much higher than negative control; dose-response relationship
	Comet test	No statistically significant differences between the samples and the negative control (*p* > 0.05)	Statistically significant differences between the samples and the negative control (*p* < 0.05)	Statistically significant differences between the samples and the negative control (*p* < 0.05, *p* < 0.01, *p* < 0.001) and effects much higher than negative control; dose-response relationship
**Carcinogenicity**	In vitro cell transformation assay (CTA)	No statistically significant differences between the samples and the negative control (*p* > 0.05)	Statistically significant differences between the samples and the negative control (*p* < 0.05)	Number of foci of the sample comparable to the positive control, statistically significant differences between the samples and the negative control (*p* < 0.05, *p* < 0.01); effects much higher than negative control
	Tumor promotion	Inhibition of the sample equivalent to the negative control	Inhibition of the sample equivalent to 50% of the positive control	Inhibition of the sample equivalent to the positive control

**Table 2:** Enrichment factors (EF) and volumes for the calculation of the dilution factors (DF).

Bioassay	EF_SPE,WWTPA_ (-)		EF_SPE,WWTPB_ (-)				V_bioassay_ (mL)	V_extract employed_ (μL)
			Grape Harvest Time		Routine Period			
	Influent Sample	Effluent Sample	Influent Sample	Effluent Sample	Influent Sample	Effluent Sample		
**Neutral Red uptake assay**	20,000	20,000	20,000	20,000	20,000	20,000	2.4	2.4; 24; 240
**ERE-tk_Luc_MCF-7 test**	20,000	20,000	20,000	20,000	20,000	20,000	1	1
**Ames test**	20,000	13,333	27,778	27,778	13,333	13,333	2	0.5; 2.5; 5; 12.5; 25.5; 50 0.75; 3.75; 7.5; 18.75; 37.5; 75 0.036; 0.36;1.8; 3.6; 9;18; 36;72
**Comet test**	20,000	13,333	27,778	27,778	13,333	13,333	1	0.05; 0.5; 2.5; 5; 25 0.08; 0.75; 3.75; 7.5; 37.5 0.04; 0.36; 1.80; 3.6; 18
**In vitro cell transformation assay (CTA)**	-	-	200,000	200,000	-	-	8	0.25; 0.5; 1.25
**Tumor promotion**	20,000	20,000	20,000	20,000	20,000	20,000	4	1

**Table 3:** Main influent and effluent wastewater characteristics measured during the monitoring campaign (average values and standard deviation, between brackets, in mg/L).

Parameter	Influent Concentration	Effluent Concentration
COD	500 (45)	30 (5.0)
BOD_5_	300 (23)	5.4 (1.2)
Total Nitrogen	74 (8.1)	19 (2.1)
Phosphorus	7.0 (1.0)	1.9 (0.2)
Total suspended solids	370 (41)	7.0 (2.0)

**Table 4:** Metals and semimetals effluent concentrations measured during the monitoring campaign (average values, in μg/L).

Parameter	Concentration	EC_50_ *D. magna* (48 h)
Boron	119	141,000 [39]
Vanadium	1.5	1200 [40]
Chromium III	13	6790 [41]
Manganese	23	9300 [40]
Iron	249	2300 [40]
Nickel	36	650 [40]
Copper	8.1	13 [40]
Selenium	0.37	710 [40]
Arsenic	0.85	2400 [40]
Cadmium	0.07	3.60 [40]
Antimony	1	4100 [40]
Aluminum	175	3900 [40]
Mercury	0.30	0.65 [40]
Lead	6.1	290 [40]
Zinc	75	720 [40]

**Table 5:** Polynuclear aromatic hydrocarbons, perfluorinated alkyl substances, chlorinated insecticides and herbicides resulted above the respected detection limits in the effluent wastewater (average values, in ng/L).

Parameter	Concentration
PFHxA (Pefluorohexanoic acid)	8.5
PFOA (Perfluoroctanoic acid) Linear	5.4
PFOA (Linear + branched isomers)	5.4
Sum of PFOA and PFOS (including linear + branched isomers)	5.4
Other PFAAs (PFBA, PFBS, PFPeA, PFHxA, PFHxS, PFHpA, PFNA, PFDeA, PFUnA, PFDoA)	8.5

**Table 6:** Results of Ames test on Salmonella typhimurium (TA98 and TA100 strain ±S9) expressed as mutagenicity ratio (MR) and REF values.

Sample	REF	MR TA98	MR TA98 +S9	MR TA100	MR TA100 +S9
**Influent sample**	5	1.0	1.0	0.7	1.0
	25	0.9	0.9	0.2	0.6
	50	0.9	0.3	tox	tox
	125	1.4	tox	tox	tox
	250	tox	tox	tox	tox
	500	tox	tox	tox	tox
**Effluent sample**	5	1.1	1.0	1.2	1.1
	25	1.3	1.2	1.5	1.3
	50	1.5	1.1	1.3	1.2
	125	0.5	0.8	0.4	0.3
	250	tox	0.2	tox	tox
	500	tox	tox	tox	tox

**Table 7:** “Traffic-light” representation of results for WWTP A. The REF value or type of sample (RU = raw undiluted, RD = raw diluted) used to establish the color of each assay are specified.

Mode of Toxic Action	Bioassay	IN	OUT
Baseline toxicity	*D. magna* test (48 h)	RU	RU
	*D. magna* test (24 h)	RU	RU
	*Aliivibrio fischeri* test	RU	RU
	*R. subcapitata* test	RU	RU
	*Allium cepa* toxicity test	RU	RU
	Neutral Red uptake assay	1	1
Endocrine disruption	ERE-tk_Luc_MCF-7 test	20	20
Genetic toxicity	Ames test	5–500	5–500
	Comet test	1–500	1–500
	*Allium cepa* genotoxicity test (CA)	RD up to 1:100	RU and RD up to 1:100
	*Allium cepa* genotoxicity test (MN)	RU and RD (1:2)	RU and RD (1:2)
Carcinogenicity	CTA	-	-
	Tumor promotion	5	5

**Table 8:** Main influent and effluent wastewater characteristics measured during both monitoring campaigns (average values and standard deviation, between brackets, in mg/L).

Parameter	Grape Harvest Time		Routine Period	
	Influent Concentration	Effluent Concentration	Influent Concentration	Effluent Concentration
COD	380 (42)	24 (2.2)	400 (51)	25 (1.4)
BOD_5_	200 (15)	12 (0.4)	170 (15)	5.0 (0.1)
Total Nitrogen	15 (2.0)	5.1 (0.2)	29 (2.5)	5.7 (0.1)
Phosphorus	2.8 (0.3)	0.2 (0.1)	3.7 (0.2)	0.3 (0.05)
Total suspended solids	180 (22)	16 (1.5)	170 (18)	10 (1.0)

**Table 9:** Metals and semimetals effluent concentrations measured during both monitoring campaigns (average values, in μg/L).

Parameter	Composite Effluent Sample Concentration		EC_50_ *D. magna* (48 h)
	Grape Harvest Time	Routine Period	
Boron	104	87	141,000 [39]
Vanadium	1.7	2.5	1200 [40]
Chromium III	5.4	<5	6790 [41]
Manganese	1.6	3.1	9300 [40]
Iron	90	79	2300 [40]
Nickel	11	7	650 [40]
Copper	4.7	4.2	13 [40]
Arsenic	1.1	1.0	2400 [40]
Selenium	<0.5	<0.5	710 [40]
Cadmium	<0.5	<0.5	3.60 [40]
Antimony	<1.0	<1.0	4100 [40]
Aluminium	109	96.1	3900 [40]
Mercury	0.28	0.27	0.65 [40]
Lead	5.9	5.1	290 [40]

**Table 10:** Polynuclear aromatic hydrocarbons, perfluorinated alkyl substances, chlorinated insecticides and herbicides resulted above the respected detection limits in the effluent wastewater (average values, in ng/L).

Parameter	Concentration	
	Grape Harvest Time	Routine Period
Metolachlor	<10	27
Terbutryn	23	17
PFHxA (Pefluoroexanoic acid)	14	16
PFOA (Perfluoroctanoic acid) Linear	22	27
PFOA (Perfluoroctanoic acid branched isomers)	7.9	8.7
PFOA (Linear + branched isomers)	30	36
PFBS (Perfluorobutansulfonate)	10	11
Sum of PFOA and PFOS (including linear + branched isomers)	30	36
Other PFAAs (PFBA, PFBS, PFPeA, PFHxA, PFHxS, PFHpA, PFNA, PFDeA, PFUnA, PFDoA)	24	27

**Table 11:** Results of Ames test on *Salmonella typhimurium* TA98 and TA100 strain ±S9 expressed as mutagenicity ratio (MR) and REF values.

Sample	REF	Mutagenicity Ratio TA98	Mutagenicity Ratio TA98 +S9	Mutagenicity Ratio TA100	Mutagenicity Ratio TA100 +S9
Influent sample (grape harvest time)	0.5	0.7	1.3	-	-
	25	0.7	0.4	1.1	1.1
	50	0.6	0.5	1.0	1.0
	125	tox	tox	tox	tox
	250	tox	tox	tox	tox
	500	tox	tox	tox	tox
	1000	tox	tox	tox	tox
Effluent sample (grape harvest time)	0.5	1.1	1.3	-	-
	25	1.5	1.1	1.2	1.1
	50	1.7	1.5	1.3	1.3
	125	0.8	0.7	1.1	1.0
	250	tox	tox	tox	tox
	500	tox	tox	tox	tox
	1000	tox	tox	tox	tox
Influent sample (routine period)	5	1.1	1.0	0.9	1.3
	25	0.9	1.0	1.2	0.9
	50	0.9	0.8	0.9	0.4
	125	tox	tox	tox	0.2
	250	tox	tox	tox	tox
	500	tox	tox	tox	tox
Effluent sample (routine period)	5	1.1	1.3	1.0	1.2
	25	2.2	1.5	1.5	1.2
	50	2.3	1.6	1.5	0.8
	125	0.7	0.5	tox	tox
	250	tox	0.5	tox	tox
	500	tox	tox	tox	tox

tox = toxic to bacteria.

**Table 12:** “Traffic-light” representation of results for WWTP B. The REF value or type of sample (RU = raw undiluted, RD = raw diluted) used to establish the color of each assay are specified.

Mode of Toxic Action	Bioassay	First Campaign (Grape Harvest Time)		Second Campaign (Routine Period)	
		IN	OUT	IN	OUT
Baseline toxicity	*D. magna* test (48 h)	RU	RU	RU	RU
	*D. magna* test (24 h)	RU	RU	RU	RU
	*Aliivibrio fischeri* test	RU	RU	RU	RU
	*R. subcapitata* test	RU	RU	RU	RU
	*Allium cepa* toxicity test	RU	RU	RU	RU
	Neutral Red uptake assay	1	1	1	1
Endocrine disruption	ERE-tk_Luc_MCF-7 test	20	20	20	20
Genetic toxicity	Ames test	0.5–1000	0.5–1000	5–500	5–500
	Comet test	1–500	1–500	1–500	1–500
	*Allium cepa* genotoxicity test (CA)	RU and RD up to 1:100	RU and RD up to 1:100	RU and RD up to 1:100	RU and RD up to 1:100
	*Allium cepa* genotoxicity test (MN)	RU	RU	RU	RU
Carcinogenicity	CTA	6–13	6 - 31	-	-
	Tumor promotion	5	5	5	5

## Supplementary Materials

The following are available online at https://www.mdpi.com/article/10.3390/ijerph18136827/s1, Figure S1: Scheme of the water line and sampling points of the WWTP A and WWTP B., Figure S2: Onions roots length (expressed as average value, in cm) of undiluted and diluted samples used to identify the concentration for the execution of Allium cepa genotoxicity assays. Figure S3: Onions roots length (expressed as average value, in cm) of undiluted and diluted samples used to identify the concentration for the execution of Allium cepa genotoxicity assays. Table S1: Origin of raw wastewater, capacity of each WWTP and treatment units., Table S2: Analytical methods for wastewater chemical characterisation. Table S3: WWTP A: values of polynuclear aromatic hydrocarbons, poly-and perfluorinated substances, chlorinated insecticides and herbicides measured in the effluent during the monitoring campaign. Table S4: WWTP A: mobility inhibition of freshwater cladoceran, growth inhibition of the unicellular green alga and reduction of the natural bioluminescence of marine bacteria (expressed as effect percentage, in %). Table S5: WWTP A: onion roots length (expresses as average value, in cm) exposed for 76 h in darkness to undiluted and diluted samples. The red value is the concentration that cause a 50% reduction in root growth (EC50). Table S6: WWTP A: 17-estradiol dose-response calibration data extrapolated by the endocrine disruption effect (expressed as the ratio between activity of luciferase and the mass of proteins). The orange rows are the values excluded by the logistic regression. Table S7: WWTP A: results of comet assay on human leukocyte, expressed as visual score and tail intensity, and REF values. Table S8: WWTP A: results of Allium cepa genotoxicity assays expressed as percentage of micronucleus respect to the negative control. Table S9: WWTP A: results of Allium cepa genotoxicity assays expressed as percentage of chromosome aberration respect to the negative control. Table S10: WWTP B: values of polynuclear aromatic hydrocarbons, chlorinated insecticides and herbicides measured in the effluent during the monitoring campaign. Table S11: WWTP B: mobility inhibition of freshwater cladoceran, growth inhibition of the unicellular green alga and reduction of the natural bioluminescence of marine bacteria (expressed as effect percentage, in %). Table S12: WWTP B: onions roots length (expresses as average value, in cm) exposed for 76 h in darkness to undiluted and diluted samples for each monitoring campaign. The red value is the concentration that cause a 50% reduction in root growth (EC50). Table S13: WWTP B: 17-estradiol dose-response calibration data extrapolated by the endocrine disruption effect (expressed as the ratio between activity of luciferase and the mass of proteins). The orange rows are the values excluded by the logistic regression. Table S14: WWTP B: results of comet assay on human leukocyte, expressed as visual score and tail intensity, and REF values. Table S15: WWTP B: results of Allium cepa genotoxicity assays expressed as percentage of micronucleus respect to the negative control in both monitoring campaigns. Table S16: WWTP B: results of Allium cepa genotoxicity assays expressed as percentage of chromosome aberration respect to the negative control in both monitoring campaigns.

## References

[1] Logar I., Brouwer R., Maurer M., Ort C. (2014). Cost-Benefit Analysis of the Swiss National Policy on Reducing Micropollutants in Treated Wastewater. Environ. Sci. Technol..

[2] Papa M., Pedrazzani R., Bertanza G. (2013). How green are environmental technologies? A new approach for a global evaluation: The case of WWTP effluents ozonation. Water Res..

[3] Papa M., Alfonsín C., Moreira M.T., Bertanza G. (2016). Ranking wastewater treatment trains based on their impacts and benefits on human health: A “Biological Assay and Disease” approach. J. Clean. Prod..

[4] Bertanza G., Papa M., Pedrazzani R., Repice C., Mazzoleni G., Steimberg N., Feretti D., Ceretti E., Zerbini I. (2013). EDCs, estrogenicity and genotoxicity reduction in a mixed (domestic+textile) secondary effluent by means of ozonation: A full-scale experience. Sci. Total. Environ..

[5] Papa M., Ceretti E., Viola G.C.V., Feretti D., Zerbini I., Mazzoleni G., Steimberg N., Pedrazzani R., Bertanza G. (2016). The assessment of WWTP performance: Towards a jigsaw puzzle evaluation?. Chemosphere.

[6] Ragazzo P., Feretti D., Monarca S., Dominici L., Ceretti E., Viola G., Piccolo V., Chiucchini N., Villarini M. (2017). Evaluation of cytotoxicity, genotoxicity, and apoptosis of wastewater before and after disinfection with performic acid. Water Res..

[7] Kunz P.Y., Simon E., Creusot N., Jayasinghe B.S., Kienle C., Maletz S., Schifferli A., Schönlau C., Aït-Aïssa S., Denslow N.D. (2017). Effect-based tools for monitoring estrogenic mixtures: Evaluation of five in vitro bioassays. Water Res..

[8] Pedrazzani R., Cavallotti I., Bollati E., Ferreri M., Bertanza G. (2018). The role of bioassays in the evaluation of ecotoxicological aspects within the PEF/OEF protocols: The case of WWTPs. Ecotoxicol. Environ. Saf..

[9] Pedrazzani R., Ziliani E., Cavallotti I., Bollati E., Ferreri M., Bertanza G. (2019). Use of ecotoxicology tools within the environmental footprint evaluation protocols: The case of wastewater treatment plants. DESALINATION Water Treat..

[10] Toxicology Testing in the 21st Century (Tox21)|Safer Chemicals Research|US EPA. https://www.epa.gov/chemical-research/toxicology-testing-21st-century-tox21.

[11] EU-ToxRisk—EU-ToxRisk—An Integrated European ‘Flagship’ Programme Driving Mechanism-based Toxicity Testing and Risk Assessment for the 21st Century. http://www.eu-toxrisk.eu/.

[12] Effectopedia—Just another WordPress Site. https://effectopedia.org/.

[13] SeqAPASS Login|US EPA. https://seqapass.epa.gov/seqapass/.

[14] Pedrazzani R., Bertanza G., Brnardić I., Cetecioglu Z., Dries J., Dvarionienė J., García-Fernández A.J., Langenhoff A., Libralato G., Lofrano G. (2019). Opinion paper about organic trace pollutants in wastewater: Toxicity assessment in a European perspective. Sci. Total. Environ..

[15] Coes A.L., Paretti N.V., Foreman W.T., Iverson J.L., Alvarez D.A. (2014). Sampling trace organic compounds in water: A comparison of a continuous active sampler to continuous passive and discrete sampling methods. Sci. Total. Environ..

[16] Aymerich I., Acuña V., Ort C., Rodríguez-Roda I., Corominas L. (2017). Fate of organic microcontaminants in wastewater treatment and river systems: An uncertainty assessment in view of sampling strategy, and compound consumption rate and degradability. Water Res..

[17] Ort C., Lawrence M.G., Reungoat J., Mueller J.F. (2010). Sampling for PPCPs in Wastewater Systems: Comparison of Different Sampling Modes and Optimization Strategies. Environ. Sci. Technol..

[18] Ort C., Lawrence M.G., Rieckermann J., Joss A. (2010). Sampling for Pharmaceuticals and Personal Care Products (PPCPs) and Illicit Drugs in Wastewater Systems: Are Your Conclusions Valid? A Critical Review. Environ. Sci. Technol..

[19] Bertanza G., Pedrazzani R., Grande M.D., Papa M., Zambarda V., Montani C., Steimberg N., Mazzoleni G., Di Lorenzo D. (2011). Effect of biological and chemical oxidation on the removal of estrogenic compounds (NP and BPA) from wastewater: An integrated assessment procedure. Water Res..

[20] Roberta P., Pietro B., Donatella F., Giovanna M., Nathalie S., Chiara U., Gaia V., Ilaria Z., Roy K. (2020). Methodological Protocol for Assessing the Environmental Footprint by Means of Ecotoxicological Tools: Wastewater Treatment Plants as an Example Case. Ecotoxicological QSARs.

[21] International Organization for Standardization (2012). Water Quality—Fresh Water Algal Growth Inhibition Test with Unicellular Green Algae.

[22] International Organization for Standardization (2007). Water Quality—Determination of the Inhibitory Effect of Water Samples on the Light Emission of Vibrio Fischeri (Luminescent Bacteria Test)—Part 3: Method Using Freeze-Dried Bacteria.

[23] International Organization for Standardization (2012). Water Quality—Determination of the Inhibition of the Mobility of Daphnia Magna Straus (Cladocera, Crustacea)—Acute Toxicity Test.

[24] Fiskesjö G. (1995). Allium Test. In Vitro Toxicity Testing Protocols.

[25] Borenfreund E., Puerner J.A. (1985). A simple quantitative procedure using monolayer cultures for cytotoxicity assays (HTD/NR-90). J. Tissue Cult. Methods.

[26] APHA (2017). Standard Methods for the Examination of Water and Wastewater.

[27] Maron D.M., Ames B.N. (1983). Revised methods for the Salmonella mutagenicity test. Mutat. Res. Mutagen. Relat. Subj..

[28] Cabaravdic M. (2010). Induction of chromosome aberrations in the Allium cepa test system caused by the exposure of cells to benzo(a) pyrene. Med. Arh..

[29] Ma T.-H., Xu Z., Xu C., McConnell H., Rabago E.V., Arreola G.A., Zhang H. (1995). The improved Allium/Vicia root tip micronucleus assay for clastogenicity of environmental pollutants. Mutat. Res. Mutagen. Relat. Subj..

[30] Tice R.R., Agurell E., Anderson D., Burlinson B., Hartmann A., Kobayashi H., Miyamae Y., Rojas E., Ryu J.-C., Sasaki Y.F. (2000). Single cell gel/comet assay: Guidelines for in vitro and in vivo genetic toxicology testing. Environ. Mol. Mutagen..

[31] Singh N.P., McCoy M.T., Tice R.R., Schneider E.L. (1988). A simple technique for quantitation of low levels of DNA damage in individual cells. Exp. Cell Res..

[32] Urani C., Stefanini F., Bussinelli L., Melchioretto P., Crosta G. (2009). Image analysis and automatic classification of transformed foci. J. Microsc..

[33] Forcella M., Callegaro G., Melchioretto P., Gribaldo L., Frattini M., Stefanini F., Fusi P., Urani C. (2016). Cadmium-transformed cells in the in vitro cell transformation assay reveal different proliferative behaviours and activated pathways. Toxicol. Vitr..

[34] (2007). Detailed Review Paper on Cell Transformation Assays for Detection of Chemical Carcinogens.

[35] El-Fouly M.H., Trosko J.E., Chang C.-C. (1987). Scrape-loading and dye transfer: A rapid and simple technique to study gap junctional intercellular communication. Exp. Cell Res..

[36] Escher B.I., Allinson M., Altenburger R., Bain P., Balaguer P., Busch W., Crago J., Denslow N.D., Dopp E., Hilscherova K. (2014). Benchmarking Organic Micropollutants in Wastewater, Recycled Water and Drinking Water with In Vitro Bioassays. Environ. Sci. Technol..

[37] Escher B.I., Neale P.A., Leusch F.D. (2015). Effect-based trigger values for in vitro bioassays: Reading across from existing water quality guideline values. Water Res..

[38] DECRETO LEGISLATIVO 3 aprile 2006, n. 152—Normattiva. https://www.normattiva.it/uri-res/N2Ls?urn:nir:stato:decreto.legislativo:2006-04-03;152.

[39] Maier K.J., Knight A.W. (1991). The toxicity of waterborne boron toDaphnia magna andChironomus decorus and the effects of water hardness and sulfate on boron toxicity. Arch. Environ. Contam. Toxicol..

[40] Okamoto A., Yamamuro M., Tatarazako N. (2015). Acute toxicity of 50 metals toDaphnia magna. J. Appl. Toxicol..

[41] Puerari R.C., Da Costa C.H., Vicentini D.S., Fuzinatto C.F., Melegari S.P., Schmidt É.C., Bouzon Z.L., Matias W.G. (2016). Synthesis, characterization and toxicological evaluation of Cr_2_O_3_ nanoparticles using Daphnia magna and Aliivibrio fischeri. Ecotoxicol. Environ. Saf..

[42] Directive 2000/60/EC of the European Parliament and of the C.—EUR-Lex. https://eur-lex.europa.eu/legal-content/EN/LSU/?uri=celex%3A32000L0060.

[43] Guidelines for Drinking-Water Quality, 4th Edition, Incorporating the 1st Addendum. https://www.who.int/publications/i/item/9789241549950.

[44] Pedrazzani R., Ceretti E., Zerbini I., Casale R., Gozio E., Bertanza G., Gelatti U., Donato F., Feretti D. (2012). Biodegradability, toxicity and mutagenicity of detergents: Integrated experimental evaluations. Ecotoxicol. Environ. Saf..

[45] Ohe T., Watanabe T., Wakabayashi K. (2004). Mutagens in surface waters: A review. Mutat. Res. Mutat. Res..

