# Supplementary material for: Environmental Footprint of Wastewater Treatment: A Step Forward in the Use of Toxicological Tools
Source: Int J Environ Res Public Health. 2021 Jun 25;18(13):6827. doi: 10.3390/ijerph18136827 (PMC8297069; doi:10.3390/ijerph18136827)
Supplement: Supplementary file 1 [file ijerph-18-06827-s001.zip › ijerph-1236345-supplementary.pdf]

Article

Environmental Footprint of Wastewater Treatment: A Step Forward in the Use of Toxicological Tools

Giorgio Bertanza [1,2], Jennifer Boniotti [3], Elisabetta Ceretti [4], Donatella Feretti [2,4], Giovanna Mazzoleni [2,3], Michele Menghini [1], Roberta Pedrazzani [2,5,*], Nathalie Steimberg [2,3], Chiara Urani [2,6], Gaia Claudia Viviana Viola [4], Ilaria Zerbini [4] and Emanuele Ziliani [7]

1 DICATAM—Department of Civil, Environmental, Architectural Engineering and Mathematics, University of Brescia, Via Branze 43, I-25123 Brescia, Italy; giorgio.bertanza@unibs.it (G.B.); m.menghini@studenti.unibs.it (M.M.)
2 MISTRAAL Interdepartmental University Research Center—MISTRAL—Integrated Study Models for the Protection of Health and Prevention in Life and Work Environments, DSCS, Department of Clinical and Experimental Sciences, University of Brescia, Viale Europa 11, I-25123 Brescia, Italy; donatella.feretti@unibs.it (D.F.); giovanna.mazzoleni@unibs.it (G.M.); nathalie.steimberg@unibs.it (N.S.); chiara.urani@unimib.it (C.U.)
3 DSCS—Department of Clinical and Experimental Sciences, University of Brescia, Viale Europa 11, I-25123 Brescia, Italy; jenniferboniotti@yahoo.it
4 DSMC—Department of Medical and Surgical Specialities, Radiological Sciences and Public Health, University of Brescia, Viale Europa 11, I-25123 Brescia, Italy; elisabetta.ceretti1@unibs.it (E.C.); gaia.viola@unibs.it (G.C.V.V.); ilaria.zerbini@unibs.it (I.Z.)
5 DIMI—Department of Mechanical and Industrial Engineering, University of Brescia, Via Branze 38, I-25123 Brescia, Italy
6 DISAT—Department of Earth and Environmental Sciences, University of Milan—Bicocca, Piazza della Scienza 1, I-20126 Milano, Italy
7 DICAr—Department of Civil Engineering and Architecture, University of Pavia, Via Ferrata 1, I-27100 Pavia, Italy; e.ziliani001@studenti.unibs.it
* Correspondence: roberta.pedrazzani@unibs.it; Tel.: +39-030-371-5505; Fax: +39-030-370-2448

SUPPLEMENTARY MATERIAL

1 aDefinition of the “traffic light” thresholds

Daphnia magna test

The results of this test, carried out according to the procedures displayed in ISO 6341:2012, can be classified as reported below:

- Green colour: the organism immobilization percentage is lower than 10%.
- Yellow colour: the organism immobilization percentage is between 10% and 50%.
- Red colour: the organism immobilization percentage is higher than 50%.

Aliivibrio fischeri test

The results of this test, carried out according to the procedures displayed in ISO 11348-3:2007, can be classified as reported below:

- Green colour: the bacterial luminescence inhibition is lower than 10%.
- Yellow colour: the bacterial luminescence inhibition is between 10% and 50%.
- Red colour: the bacterial luminescence inhibition is higher than 50%.

Raphidocelis subcapitata test

The results of this test, carried out according to the procedures displayed in ISO 8692:2012, can be classified as reported below:

- Green colour: the growth inhibition is lower than 10%.
- Yellow colour: the growth inhibition is between 10% and 50%.
- Red colour: the growth inhibition is higher than 50%.

Allium cepa toxicity test

Roots length of *Allium cepa* germinated in undiluted and diluted wastewater is used to calculate the EC_{50} (the effect concentration causing 50% growth root reduction in comparison with the control) and to identify the concentration to be used in the *A. cepa* genotoxicity assays. Other macroscopic parameters (turgescence, consistency, root form, change in colour, root tip shape) are used as toxicity indexes (Fiskesjö, 1995). Numerous dilutions have been used: 1:2, 1:10, 1:20, 1:100, 1:200, 1:1000.

The following criteria have been adopted to associate the colours of the traffic light with the results:

- Green colour: the length of the roots in wastewater is longer than 50% roots length in the negative control (undiluted sample $< EC_{50}$).
- Yellow colour: the length of the roots in undiluted wastewater is equal to 50% roots length in the negative control (undiluted sample $= EC_{50}$).
- Red colour: the length of the roots in undiluted wastewater is shorter than 50% roots length in the negative control (undiluted sample $> EC_{50}$).

Neutral Red uptake assay

This test allows to determine cell viability determined by spectrophotometry. Results are expressed as a percentage of death cells in treated vs untreated cells and are used to extrapolate a dose-response curve (the experimental results were normalized in order to achieve the zero effect corresponding to the negative control effect and the maximum effect at the highest tested dose). Cells were treated with REF 20, 200, 2000 for either 24 or 48 hours.

In our conditions, the EC_{50} (the concentration of a solution inducing a cell response resulting in the diminution of cell number by half) was determined for each time of treatment.

The following correspondence has been considered:

- Green colour: the sample with a REF of 1 induce less than 20% loss of cells (measured effect $< EC_{20}$).

- Yellow colour: the sample with a REF of 1 induced a loss of cells ranging from 20% to 50%, the cytotoxicity effect is concrete (EC_{20} < measured effect < EC_{50}).
- Red colour: the sample with a REF of 1 induce a loss of cells higher than 50%, the treatment is considered highly toxic (measured effect > EC_{50}).

ERE-tk_Luc_MCF-7 test

17β-Estradiol (E2) is used to obtain standard curve (the experimental results were normalized in order to achieve the zero effect corresponding to the negative control effect and the maximum effect at the highest tested dose) for luciferase activity as a function of E2 concentration. Results obtained with the different concentrations of E2 help to determine the E2 equivalent concentration corresponding to the estrogenic activity expressed in our sample.

The following threshold have been adopted:

- Green colour: luciferase activity induced by sample is lower than the 20% of the maximum effect obtainable with E2;
- Yellow colour: luciferase activity induced by the sample is ranging between the 20 and 50 % of the maximum effect obtainable with E2;
- Red colour: luciferase activity induced by the sample is higher than the 50% of the maximum effect obtainable with E2.

Ames test

The Ames test allows to detect point mutations, i.e. substitution of bases and frame-shift mutations, in bacteria. The Ames test results are expressed as the mutagenicity ratio (MR), obtained by dividing the revertants induced by the sample by the spontaneous mutation rate (revertants induced by the negative/solvent control). The results are considered positive when a reproducible, dose-related increase in the number of revertant colonies in one or more strains is observed. More in detail the test is positive if two consecutive dose levels, or the highest nontoxic dose level, produce a response at least twice that of the solvent control and at least two of these consecutive doses show a dose-response relationship (APHA, 2017; Maron and Ames, 1983). Moreover, the results are expressed as specific mutagenic activity, i.e. induced revertants/L_{eq} of tested wastewater, calculated by linear regression analysis on the dose-response curves. Net revertants may be calculated only in the presence of dose-response curve.

According to Ohe et al. (2004), the number of revertants per equivalent liter has been taken into account. Therefore, the quality intervals have been expressed as a function of the mutagenicity ratio.

The following thresholds have been adopted:

- Green colour: mutagenicity ratio lower than 2.
- Yellow colour: mutagenicity ratio in between 2 and 2.5.
- Red colour: mutagenicity ratio higher than 2.5.

Allium cepa genotoxicity tests

Allium cepa genotoxicity test allows to detect chromosomal mutations in plant cells. Allium cepa genotoxicity assays are considered positive according to statistical evaluation: chi-square and Dunnett's tests for chromosome aberrations (CA) and micronuclei (MN) frequency, respectively, are used to determine the significance of the difference between the samples and negative control at the 5% significance level (Cabaravdic, 2010; Ma et al., 1995). The results are also evaluated using the linear regression analysis to establish their correlation with doses (various dilutions).

The following correspondence has been considered:

- Green colour: there are no statistically significant differences between the samples and the negative control (statistical test negative, $p>0.05$).
- Yellow colour: there are statistically significant differences between the samples and the negative control ($p<0.05$).
- Red colour: there are statistically significant differences between the samples and the negative control ($p<0.05$, $p<0.01$, $p<0.001$), values higher than 2 times the negative control and eventually a dose-response relationship.

Single Cell Gel Electrophoresis assay (Comet test)

The comet assay allows to detect primary DNA damage in mammalian cells. The Comet test results are expressed as the extent of DNA migration evaluated by both ‘‘visual score’’ (based on visual classification of DNA damage) and the comet parameter ‘‘tail intensity’’ (percentage of DNA migration in the tail) detected using an automatic imaging system (Komet 5, Kinetic Imaging Ltd). Results of Comet assay on human leukocytes are considered positive according to statistical evaluation using univariate analyses of variance (ANOVA) and Dunnett's test at the 5% significance level (APHA, 2017; Singh et al., 1988; Tice et al., 2000). The presence of a dose-response curve is also evaluated.
As for the three levels of attention, the following criteria have been adopted:

- Green colour: there are no statistically significant differences between the samples and the negative control (statistical test negative, $p>0.05$).
- Yellow colour: there are statistically significant differences between the samples and the negative control ($p<0.05$).
- Red colour: there are statistically significant differences between the samples and the negative control ($p<0.05$, $p<0.01$, $p<0.001$), effects much higher than negative control and a dose-response relationship.

In vitro cell transformation assay (CTA)

The CTA is an *in vitro* experimental model for the identification of the carcinogenic potential of chemicals. This assay mimics key stages of the conversion of normal into transformed cells with malignant phenotypes, as in the *in vivo* multistage carcinogenesis process. C3H10T1/2 mouse embryo fibroblasts provide the advantage of high sensitivity, specificity, positive predictivity along with low negative predictivity and false positive or false negative rates (OECD, 2007; Priya et al., 2013). Endpoint of the assay is the formation of colonies of transformed cells (*foci, Type II* and *III)* classified on standard morphological features: dense basophilic staining, multi-layered growth, random orientation of the cells at the edge of *foci*, invasive growth on surrounding monolayer of contact-inhibited cells, and spindle shape of transformed cells (Urani et al., 2009). The results (number of *foci*) in treated samples are statistically compared to the negative and to the positive controls, where 3-methylcholantrene (4μg/ml) was used as a positive control.

As for the three levels of attention, the following criteria have been adopted:

- Green colour: no statistically significant differences between the number of transformed *foci* in treated samples and the negative control (statistical test negative, $p>0.05$).
- Yellow colour: there are statistically significant differences between the number of transformed *foci* in treated samples and the negative control ($p<0.05$).
- Red colour: there are statistically significant differences between the number of *foci* in treated samples and the negative control ($p<0.05$, $p<0.01$); in addition, the effects are much higher than in negative control and/or comparable to the positive control.

Tumour promotion

Lucifer dye transfer from cell to cell by mean of GJIC was evaluated by counting fluorescent cells in all experimental conditions. This test to be considered significant should take into account values similar to controls (TPA treated or untreated cells). However, when calculating the t-test of sample vs TPA and sample vs negative control the classification is easier.

The attribution of the color for the traffic light ranging system are based on the following criteria:

- Green colour: GJIC determined in cells that were not treated (control 0% inhibition of cell communication) was used as reference for absence of toxicity (No toxicity is observed).
- Yellow: GJIC inhibition about 50% was considered as noteworthy (toxicity is concrete).
- Red: GJIC determined in cells that were treated with well-known tumor promoter (TPA) was used as reference for the maximum of toxicity (100% inhibition) (Toxicity is undoubtedly demonstrated).

2 Figures

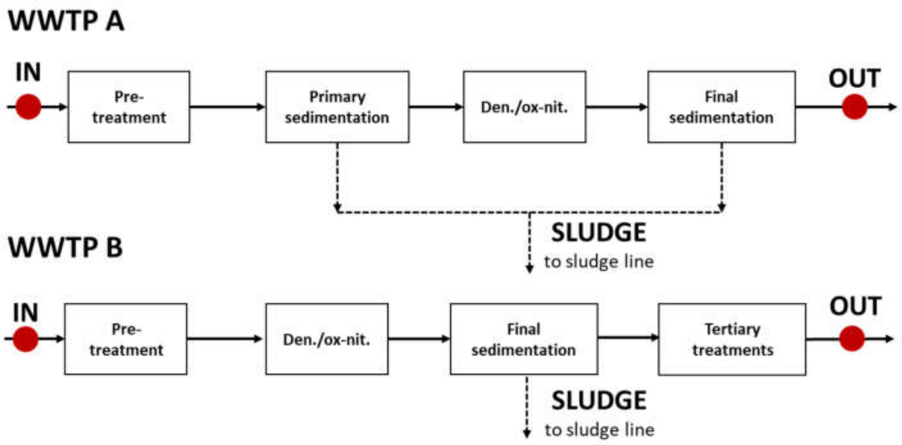

Figure S1. Scheme of the water line and sampling points of the WWTP A and WWTP B.

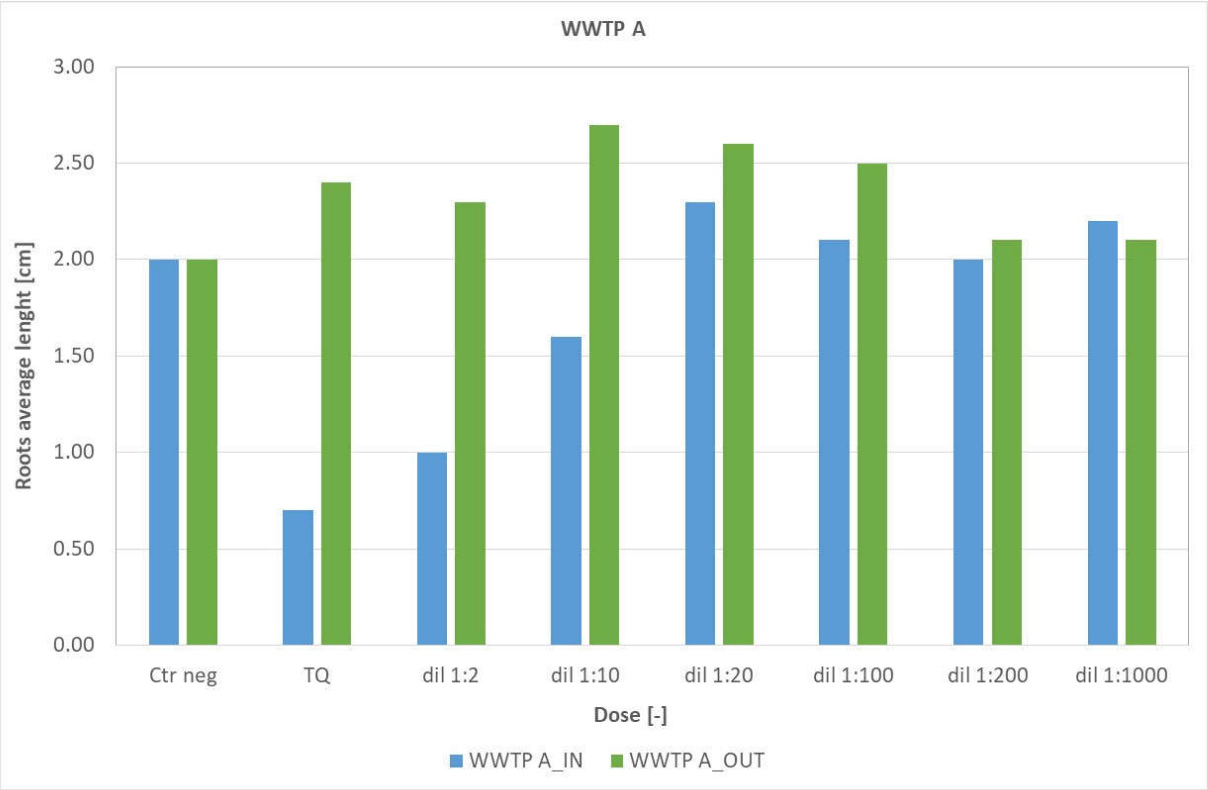

Figure S2. Onions roots length (expressed as average value, in cm) of undiluted and diluted samples used to identify the concentration for the execution of Allium cepa genotoxicity assays.

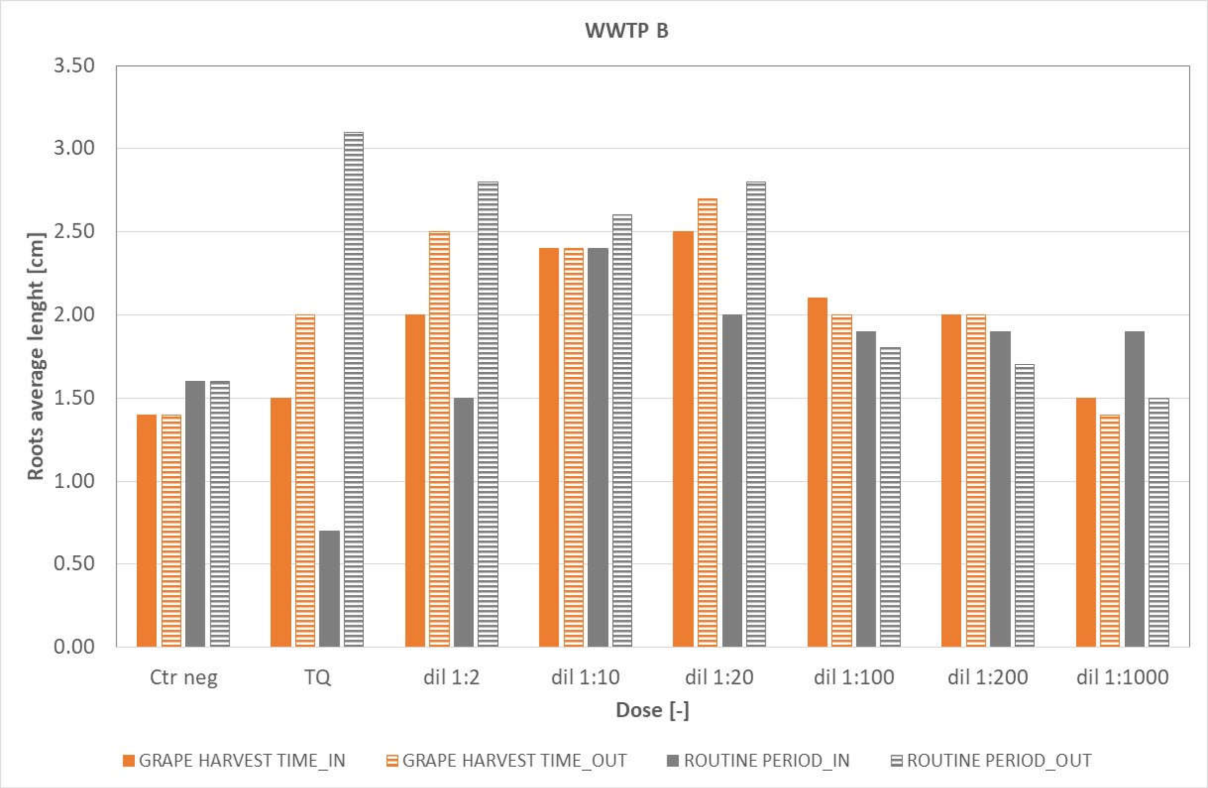

Figure S3. Onions roots length (expressed as average value, in cm) of undiluted and diluted samples used to identify the concentration for the execution of Allium cepa genotoxicity assays.

3 Tables

Table S1. Origin of raw wastewater, capacity of each WWTP and treatment units.

WWTP	Wastewater origin	Nominal capacity (p. e.)	Treatment type
**A**	domestic	370,000	SI, DN, N, SII
**B**	domestic and industrial^	60,000	DN, N, SII, F, UV

SI: primary sedimentation, DN: denitrification, N: nitrification-oxidation, SII: secondary sedimentation, F: disk filtration, UV: UV disinfection

^ winery wastewater

Table S2. Analytical methods for wastewater chemical characterisation.

**Parameter**	**Method**
Boron	EPA 6020B
Vanadiium	EPA 6020B
Chromium	EPA 6020B
Manganese	EPA 6020B
Iron	EPA 6020B
Nickel	EPA 6020B
Copper	EPA 6020B
Arsenic	EPA 6020B
Selenium	EPA 6020B
Cadmium	EPA 6020B
Antimony	EPA 6020B
Aluminium	EPA 6020B
Mercury	EPA 6020B
Lead	EPA 6020B
Alachlor	EPA 536
Ametrine	EPA 536
Atrazine	EPA 536
Cyanazine	EPA 536
Des-ethyl atrazine (DEA)	EPA 536
Desethylterbuthylazine	EPA 536
Des -sopropyl atrazine (DIA)	EPA 536
2,4-Diamino-6-chloro-s-triazine (DACT)	EPA 536
Flufenacet	EPA 536
Isoxaflutole	EPA 536
Metolachlor	EPA 536
Molinate	EPA 536
Oxadiazon	EPA 536
Pendimenthalin	EPA 536
Prometrin	EPA 536
Simazine	EPA 536
Terbuthylazine	EPA 536
Terbutryn	EPA 536
PFBA (Perfluorobutanoic Acid)	EPA 537

PFPeA (Perfluoropentanoic acid)	EPA 537
PFHxA (Pefluoroexanoic acid)	EPA 537
PFHpA (Perfluoroeptanoic acid)	EPA 537
PFOA (Perfluoroctanoic acid) Linear	EPA 537
PFOA (Perfluoroctanoic acid) branched isomers, as PFOA	EPA 537
PFOA sum of linear and branched isomers, as PFOA	EPA 537
PFNA (Perfluorononanoic acid)	EPA 537
PFDeA (Perfluorodecanoic acid)	EPA 537
PFDoA (Pefluorododecanoic acid)	EPA 537
PFUnA (Perfluoroundecanoic acid)	EPA 537
PFOS (Perfluoroctansulfonate) lineare	EPA 537
PFOS (Perfluoroctansulfonate) isohed isomers, as PFOS	EPA 537
PFOS sum of linear and branched isomers, as PFOS	EPA 537
PFBS (Perfluorobutansulfonate)	EPA 537
PFHxS (Perfluoroexansulfonate)	EPA 537
Alpha-bhc	EPA 3510 C 1996 + EPA 8270 D 2007
Beta-bhc	EPA 3510 C 1996 + EPA 8270 D 2007
Delta-bhc	EPA 3510 C 1996 + EPA 8270 D 2007
Gamma-bhc	EPA 3510 C 1996 + EPA 8270 D 2007
Heptachlor	EPA 3510 C 1996 + EPA 8270 D 2007
Aldrin	EPA 3510 C 1996 + EPA 8270 D 2007
Heptachlor Epoxide Isomer B	EPA 3510 C 1996 + EPA 8270 D 2007
Endosulfan I (alpha)	EPA 3510 C 1996 + EPA 8270 D 2007
4,4'-DDE	EPA 3510 C 1996 + EPA 8270 D 2007
Dieldrin	EPA 3510 C 1996 + EPA 8270 D 2007
Endrin	EPA 3510 C 1996 + EPA 8270 D 2007
Endosulfan II (beta)	EPA 3510 C 1996 + EPA 8270 D 2007
4,4'-DDD	EPA 3510 C 1996 + EPA 8270 D 2007
Endrin Aldehyde	EPA 3510 C 1996 + EPA 8270 D 2007
Endosulfan II (beta)	EPA 3510 C 1996 + EPA 8270 D 2007
4,4'-DDT	EPA 3510 C 1996 + EPA 8270 D 2007
Methoxychlor	EPA 3510 C 1996 + EPA 8270 D 2007
O,O,O-Triethylphosphorothioate	EPA 3510 C 1996 + EPA 8270 D 2007
Thionazin	EPA 3510 C 1996 + EPA 8270 D 2007
Sulphotep	EPA 3510 C 1996 + EPA 8270 D 2007
Forate	EPA 3510 C 1996 + EPA 8270 D 2007
Dimethoate	EPA 3510 C 1996 + EPA 8270 D 2007
Disulfoton	EPA 3510 C 1996 + EPA 8270 D 2007
Methyl Parathion	EPA 3510 C 1996 + EPA 8270 D 2007
Parathion	EPA 3510 C 1996 + EPA 8270 D 2007
Famphur	EPA 3510 C 1996 + EPA 8270 D 2007
Anthracene	EPA 3510 C 1996 + EPA 8270 D 2007
Pyrene	EPA 3510 C 1996 + EPA 8270 D 2007
Benzo[a]anthracene	EPA 3510 C 1996 + EPA 8270 D 2007
Benzo[a]pyrene	EPA 3510 C 1996 + EPA 8270 D 2007
Benzo[b]fluorantene	EPA 3510 C 1996 + EPA 8270 D 2007
Benzo[g,h,i]perylene	EPA 3510 C 1996 + EPA 8270 D 2007
Benzo[k]fluorantene	EPA 3510 C 1996 + EPA 8270 D 2007
Chrysene	EPA 3510 C 1996 + EPA 8270 D 2007
Dibenz[a,h]anthracene	EPA 3510 C 1996 + EPA 8270 D 2007
Indeno[1,2,3-cd]pyrene	EPA 3510 C 1996 + EPA 8270 D 2007

Table S3. WWTP A: values of polynuclear aromatic hydrocarbons, poly-and perfluorinated substances, chlorinated insecticides and herbicides measured in the effluent during the monitoring campaign.

Parameter	Unit	Composite effluent sample concentration
Alachlor	μg/L	< 0.01
Ametrin	μg/L	< 0.01
Atrazine	μg/L	< 0.01
Cyanazine	μg/L	< 0.01
Atrazine desethyl	μg/L	< 0.01
Desethyl terbuthylazine	μg/L	< 0.01
Desisopropyl atrazine	μg/L	< 0.01
Desethyl desisopropyl atrazine (DACT)	μg/L	< 0.01
Flufenacet	μg/L	< 0.01
Isoxaflutole	μg/L	< 0.01
Metolachlor	μg/L	< 0.01
Molinate	μg/L	< 0.01
Oxadiazon	μg/L	< 0.01
Pendimethalin	μg/L	< 0.01
Prometrine	μg/L	< 0.01
Simazine	μg/L	< 0.01
Terbuthylazine	μg/L	< 0.01
Terbutryn	μg/L	< 0.01
PFBA (Perfluorobutanoic Acid)	ng/L	< 5
PFPeA (Perfluoropentanoic acid)	ng/L	< 5
PFHxA (Pefluoroexanoic acid)	ng/L	9
PFHpA (Perfluoroeptanoic acid)	ng/L	< 5
PFOA (Perfluoroctanoic acid) Linear	ng/L	5
PFOA (Perfluoroctanoic acid branched isomers)	ng/L	< 5
PFOA (Linear + branched isomers)	ng/L	5
PFNA (Perfluorononanoic acid)	ng/L	< 5
PFDeA (Perfluorodecanoic acid)	ng/L	< 5
PFDoA (Pefluorododecanoic acid)	ng/L	< 5
PFUnA (Perfluoroundecanoic acid)	ng/L	< 5
PFOS (Perfluoroctansulfonate) linear	ng/L	< 5
PFOS (Perfluoroctansulfonate branched isomers)	ng/L	< 5
PFOS (Linear + branched isomers)	ng/L	< 5
PFBS (Perfluorobutansulfonate)	ng/L	< 5
PFHxS (Perfluoroexansulfonate)	ng/L	< 5
Sum of PFOA and PFOS (including linear + branched isomers)	ng/L	5

Other PFAAs (PFBA, PFBS, PFPeA, PFHxA, PFHxS, PFHpA, PFNA, PFDeA, PFUnA, PFDoA)	ng/L	9
Alpha-bhc	mg/L	< 0.01
Beta-bhc	mg/L	< 0.01
Delta-bhc	mg/L	< 0.01
Gamma-bhc	mg/L	< 0.01
Heptachlor	mg/L	< 0.01
Aldrin	mg/L	< 0.01
Heptachlor Epoxide Isomer B	mg/L	< 0.01
Endosulfan I (alpha)	mg/L	< 0.01
4,4'-DDE	mg/L	< 0.01
Dieldrin	mg/L	< 0.01
Endrin	mg/L	< 0.01
Endosulfan II (beta)	mg/L	< 0.01
4,4'-DDD	mg/L	< 0.01
Endrin Aldehyde	mg/L	< 0.01
Endosulfan II (beta)	mg/L	< 0.01
4,4'-DDT	mg/L	< 0.01
Methoxychlor	mg/L	< 0.01
0,0,0-Triethylphosphorothioate	mg/L	< 0.01
Thionazin	mg/L	< 0.01
Sulphotep	mg/L	< 0.01
Forate	mg/L	< 0.01
Dimethoate	mg/L	< 0.01
Disulfoton	mg/L	< 0.01
Methyl Parathion	mg/L	< 0.01
Parathion	mg/L	< 0.01
Famphur	mg/L	< 0.01
Anthracene	µg/L	< 0.01
Pyrene	µg/L	< 0.01
Benzo[a]anthracene	µg/L	< 0.01
Benzo[a]pyrene	µg/L	< 0.005
Benzo[b]fluorantene	µg/L	< 0.01
Benzo[g,h,i]perylene	µg/L	< 0.01
Benzo[k]fluorantene	µg/L	< 0.01
Chrysene	µg/L	< 0.01
Dibenz[a,h]anthracene	µg/L	< 0.01
Indeno[1,2,3-cd]pyrene	µg/L	< 0.01

Table S4. WWTP A: mobility inhibition of freshwater cladoceran, growth inhibition of the unicellular green alga and reduction of the natural bioluminescence of marine bacteria (expressed as effect percentage, in %).

**Sample**	***D.magna***		***R.subcapitata***	***A.fischeri***
	**24h**	**48h**	**72 h**	**15 min**
REF 1 influent	30	30	0	48
REF 1 effluent	15	20	11.8	56

Table S5: WWTP A: onion roots length (expresses as average value, in cm) exposed for 76 hours in darkness to undiluted and diluted samples. The red value is the concentration that cause a 50% reduction in root growth (EC_{50}).

DOSE		Onion roots length	
**DILUITION**	**(%)**	**WWTP A_IN**	**WWTP A_OUT**
**Ctr neg**	**0%**	2.0	2.0
**TQ**	**100%**	0.7	2.4
**dil 1:2**	**50%**	1.0	2.3
**dil 1:10**	**10%**	1.6	2.7
**dil 1:20**	**5%**	2.3	2.6
**dil 1:100**	**1%**	2.1	2.5
**dil 1:200**	**0,5%**	2.0	2.1
**dil 1:1000**	**0,1%**	2.2	2.1

Table S6. WWTP A: 17β-estradiol dose-response calibration data extrapolated by the endocrine disruption effect (expressed as the ratio between activity of luciferase and the mass of proteins). The orange rows are the values excluded by the logistic regression.

Concentration	Effect	Normalized effect
**[M]**	**[RLU/μg$_{proteins}$]**	**[%]**
Negative control	228353.7	0
1E-15	407694.4	-
1E-14	289215.3	13.73
1E-13	433906.3	46.37
1E-12	552361.8	73.09
1E-11	602231.2	84.34
1E-10	636998.7	92.19
1E-9	646969.7	-
1E-8	671641.4	100

Table S7. WWTP A: results of comet assay on human leukocyte, expressed as visual score and tail intensity, and REF values.

Sample	REF	Visual score	Tail intensity
**Influent sample**	1	176.0 ± 0.0**°	7.6 ± 1.7*°
	10	tox	tox
	50	tox	tox
	100	tox	tox
	500	tox	tox
**Effluent sample**	1	126.0 ± 2.8	1.0 ± 0.6
	10	146.0 ± 5.7**	3.2 ± 2.3
	50	148.0 ± 5.7**	5.0 ± 0.8*
	100	190.0 ± 5.7***	10.2 ± 0.3***
	500	tox	tox
**Negative control**	-	115.0 ± 4.2	0.6 ± 0.2

**(distilled water)**			
**Positive control (EMS 2 mM)**	-	235.0 ± 1.4	16.4 ± 5.2

*p<0.01; **p<0.05; ***p<0.001 statistically significant according to Dunnett's *t*-test vs. negative control
°p<0.001 statistically significant according to Dunnett's *t*-test vs. effluent

Table S8. WWTP A: results of Allium cepa genotoxicity assays expressed as percentage of micronucleus respect to the negative control.

**Influent**			**Effluent**		
	**IM (%)**	**MN (%)**		**IM (%)**	**MN (%)**
**TQ**	-	-	***TQ (EC_{50})***	9.5	0.11 ± 0.08
Diluition 1:2 (EC_{50})	10.2	0.06 ± 0.07	**Diluition 1:2**	9.5	0.06 ± 0.07
**Negative control**	9.7	0.05 ± 0.04	**Negative control**	9.7	0.05 ± 0.04

Table S9. WWTP A: results of Allium cepa genotoxicity assays expressed as percentage of chromosome aberration respect to the negative control.

**Influent**			**Effluent**		
	**IM (%)**	**AC (%)**		**IM (%)**	**AC (%)**
**TQ**	-	-	***TQ (EC_{50})***	10.4	4.2
Diluition 1:2 (EC_{50})	5.8	tox	**Diluition 1:2**	9.6	4.4
**Diluition 1:10**	11.1	3.9	**Diluition 1:10**	9.6	3.1
**Diluition 1:100**	12.4	4.1	**Diluition 1:100**	10.4	3.6
**Negative control**	12.4	3.5	**Negative control**	12.4	3.5

Table S10. WWTP B: values of polynuclear aromatic hydrocarbons, chlorinated insecticides and herbicides measured in the effluent during the monitoring campaign.

**Parameter**	**Unit**	**Composite effluent sample concentration**	
		**Grape harvest time**	**Routine period**
Alachlor	μg/L	< 0.01	< 0.01
Ametrin	μg/L	< 0.01	< 0.01
Atrazine	μg/L	< 0.01	< 0.01
Cyanazine	μg/L	< 0.01	< 0.01
Atrazine desethyl	μg/L	< 0.01	< 0.01
Desethyl terbuthylazine	μg/L	< 0.01	< 0.01
Desisopropyl atrazine	μg/L	< 0.01	< 0.01

Desethyl desisopropyl atrazine (DACT)	µg/L	< 0.01	< 0.01
Flufenacet	µg/L	< 0.01	< 0.01
Isoxaflutole	µg/L	< 0.01	< 0.01
Metolachlor	µg/L	< 0.01	0.027
Molinate	µg/L	< 0.01	< 0.01
Oxadiazon	µg/L	< 0.01	< 0.01
Pendimethalin	µg/L	< 0.01	< 0.01
Prometrine	µg/L	< 0.01	< 0.01
Simazine	µg/L	< 0.01	< 0.01
Terbuthylazine	µg/L	< 0.01	< 0.01
Terbutryn	µg/L	0.023	0.017
PFBA (Perfluorobutanoic Acid)	ng/L	< 5	< 5
PFPeA (Perfluoropentanoic acid)	ng/L	< 5	< 5
PFHxA (Pefluoroexanoic acid)	ng/L	14	16
PFHpA (Perfluoroeptanoic acid)	ng/L	< 5	< 5
PFOA (Perfluoroctanoic acid) Linear	ng/L	22	27
PFOA (Perfluoroctanoic acid branched isomers)	ng/L	8	9
PFOA (Linear + branched isomers)	ng/L	30	36
PFNA (Perfluorononanoic acid)	ng/L	< 5	< 5
PFDeA (Perfluorodecanoic acid)	ng/L	< 5	< 5
PFDoA (Pefluorododecanoic acid)	ng/L	< 5	< 5
PFUnA (Perfluoroundecanoic acid)	ng/L	< 5	< 5
PFOS (Perfluoroctansulfonate) linear	ng/L	< 5	< 5
PFOS (Perfluoroctansulfonate branched isomers)	ng/L	< 5	< 5
PFOS (Linear + branched isomers)	ng/L	< 5	< 5
PFBS (Perfluorobutansulfonate)	ng/L	10	11
PFHxS (Perfluoroexansulfonate)	ng/L	< 5	< 5
Sum of PFOA and PFOS (including linear + branched isomers)	ng/L	30	36
Other PFAAs (PFBA, PFBS, PFPeA, PFHxA, PFHxS, PFHpA, PFNA, PFDeA, PFUnA, PFDoA)	ng/L	24	27
Alpha-bhc	mg/L	< 0.01	< 0.01
Beta-bhc	mg/L	< 0.01	< 0.01
Delta-bhc	mg/L	< 0.01	< 0.01
Gamma-bhc	mg/L	< 0.01	< 0.01
Heptachlor	mg/L	< 0.01	< 0.01
Aldrin	mg/L	< 0.01	< 0.01
Heptachlor Epoxide Isomer B	mg/L	< 0.01	< 0.01
Endosulfan I (alpha)	mg/L	< 0.01	< 0.01
4,4'-DDE	mg/L	< 0.01	< 0.01
Dieldrin	mg/L	< 0.01	< 0.01
Endrin	mg/L	< 0.01	< 0.01
Endosulfan II (beta)	mg/L	< 0.01	< 0.01
4,4'-DDD	mg/L	< 0.01	< 0.01
Endrin Aldehyde	mg/L	< 0.01	< 0.01
Endosulfan II (beta)	mg/L	< 0.01	< 0.01
4,4'-DDT	mg/L	< 0.01	< 0.01
Methoxychlor	mg/L	< 0.01	< 0.01

0,0,0-Triethylphosphorothioate	mg/L	< 0.01	< 0.01
Thionazin	mg/L	< 0.01	< 0.01
Sulphotep	mg/L	< 0.01	< 0.01
Forate	mg/L	< 0.01	< 0.01
Dimethoate	mg/L	< 0.01	< 0.01
Disulfoton	mg/L	< 0.01	< 0.01
Methyl Parathion	mg/L	< 0.01	< 0.01
Parathion	mg/L	< 0.01	< 0.01
Famphur	mg/L	< 0.01	< 0.01
Anthracene	μg/L	< 0.01	< 0.01
Pyrene	μg/L	< 0.01	< 0.01
Benzo[a]anthracene	μg/L	< 0.01	< 0.01
Benzo[a]pyrene	μg/L	< 0.005	< 0.005
Benzo[b]fluorantene	μg/L	< 0.01	< 0.01
Benzo[g,h,i]perylene	μg/L	< 0.01	< 0.01
Benzo[k]fluorantene	μg/L	< 0.01	< 0.01
Chrysene	μg/L	< 0.01	< 0.01
Dibenz[a,h]anthracene	μg/L	< 0.01	< 0.01
Indeno[1,2,3-cd]pyrene	μg/L	< 0.01	< 0.01

Table S11. WWTP B: mobility inhibition of freshwater cladoceran, growth inhibition of the unicellular green alga and reduction of the natural bioluminescence of marine bacteria (expressed as effect percentage, in %).

PERIOD	SAMPLE	$I_{max, tq}$ [%]			
		D.magna		*R.subcapitata*	*V.fischeri*
		24h	48h	72 h	15 min
Grape harvest time	*REF 1 influent*	15	20	6.84	24
	REF 1 effluent	5	25	29.6	0
Routine period	*REF 1 influent*	10	15	15.9	18
	REF 1 effluent	5	10	13.2	0

Table S12. WWTP B: onions roots length (expresses as average value, in cm) exposed for 76 hours in darkness to undiluted and diluted samples for each monitoring campaign. The red value is the concentration that cause a 50% reduction in root growth (EC_{50}).

.DOSE		Roots average lenght [cm]			
		Grape harvest time		Routine time	
DILUITION	(%)	WWTP B_IN	WWTP B_OUT	WWTP B_IN	WWTP B_OUT
**Ctr neg**	**0%**	1.4	1.4	1.6	1.6
**TQ**	**100%**	1.5	2.0	0.7	3.1
**dil 1:2**	**50%**	2.0	2.5	1.5	2.8
**dil 1:10**	**10%**	2.4	2.4	2.4	2.6
**dil 1:20**	**5%**	2.5	2.7	2.0	2.8
**dil 1:100**	**1%**	2.1	2.0	1.9	1.8
**dil 1:200**	**0,5%**	2.0	2.0	1.9	1.7
**dil 1:1000**	**0,1%**	1.5	1.4	1.9	1.5

Table S13. WWTP B: 17β-estradiol dose-response calibration data extrapolated by the endocrine disruption effect (expressed as the ratio between activity of luciferase and the mass of proteins). The orange rows are the values excluded by the logistic regression.

**Concentration**	**Routine period**		**Grape harvest time**	
	**Effect**	**Normalized effect**	**Effect**	**Normalized effect**
**[M]**	**[RLU/μg$_{proteins}$]**	**[%]**	**[RLU/μg$_{proteins}$]**	**[%]**
Negative control	228353.7	0	204817.6	0
1E-15	407694.4	-	426856.8	-
1E-14	289215.3	13.73	309514.7	28.76
1E-13	433906.3	46.37	469062.3	72.59
1E-12	552361.8	73.09	529233.3	89.12
1E-11	602231.2	84.34	544911.2	93.43
1E-10	636998.7	92.19	568839.5	100
1E-9	646969.7	-	572851.9	-
1E-8	671641.4	100	562764.8	-

Table S14: WWTP B: results of comet assay on human leukocyte, expressed as visual score and tail intensity, and REF values.

Sample	REF	Visual score	Tail intensity
**Influent sample (grape harvest time)**	1	146.0 ± 0.0***	2.9 ± 0.1
	10	208.0 ± 0.0***°	12.4 ± 3.7*°
	50	tox	tox
	100	tox	tox
	500	tox	tox
**Effluent sample (grape harvest time)**	1	130.0 ± 2.8*	1.9 ± 0.1
	10	130.0 ± 2.8*	2.1 ± 1.6
	50	132.0 ± 0.0*	1.6 ± 0.6
	100	165.0 ± 4.2***	7.1 ± 0.3***
	500	tox	tox
**Influent sample (routine period)**	1	137.0 ± 1.4*	1.0 ± 0.0
	10	222.0 ± 5.7***°	14.9 ± 1.7***°
	50	tox	tox
	100	tox	tox
	500	tox	tox
**Effluent sample (routine period)**	1	129.0 ± 4.2	2.5 ± 1.5
	10	123.0 ± 4.2	1.6 ± 0.7
	50	147.0 ± 1.4**	2.9 ± 0.9
	100	169.0 ± 7.1***	6.8 ± 1.1***
	500	tox	tox
**Negative control (distilled water)**	-	115.0 ± 4.2	0.6 ± 0.2
**Positive control (EMS 2 mM)**	-	235.0 ± 1.4	16.4 ± 5.2

*p<0.01; **p<0.05; ***p<0.001 statistically significant according to Dunnett's *t*-test vs. negative control
°p<0.001 statistically significant according to Dunnett's *t*-test vs. effluent

Table S15. WWTP B: results of Allium cepa genotoxicity assays expressed as percentage of micronucleus respect to the negative control in both monitoring campaigns.

	IM (%)	MN (%)		IM (%)	MN (%)
**Influent (grape harvest time)**			**Effluent (grape harvest time)**		
**TQ (EC50)**	9.9	0.08 ± 0.0	**TQ (EC50)**	11.0	0.03 ± 0.01
**Influent (routine period)**			**Influent (routine period)**		
**TQ (EC50)**	11.9	0.14 ± 0.10	**TQ (EC50)**	9.2	0.12 ± 0.13

Table S16. WWTP B: results of Allium cepa genotoxicity assays expressed as percentage of chromosome aberration respect to the negative control in both monitoring campaigns.

	IM (%)	AC (%)		IM (%)	AC (%)
**Influent (grape harvest time)**			**Effluent (grape harvest time)**		
**TQ (EC50)**	8.8	4.4*°	**TQ (EC50)**	9.6	3.3
**Diluition 1:2**	10.3	3.4	**Diluition 1:2**	11.0	2.8
**Diluition 1:10**	11.9	3.5	**Diluition 1:10**	10.2	2.8
**Diluition 1:100**	10.3	3.6	**Diluition 1:100**	11.0	3.2
**Negative control**	10.7	2.5	**Negative control**	10.7	2.5
**Influent (routine period)**			**Influent (routine period)**		
**TQ (EC50)**	10.6	2.9	**TQ (EC50)**	9.0	4.1
**Diluition 1:2**	8.3	4.6	**Diluition 1:2**	11.1	3.3
**Diluition 1:10**	10.7	4.8	**Diluition 1:10**	10.5	3.5
**Diluition 1:100**	8.8	4.5	**Diluition 1:100**	10.7	3.9
**Negative control**	10.0	3.5	**Negative control**	10.0	3.5

* $p<0.001$ vs negative control (distilled water); °$p<0.001$ vs. effluent
